# Assessment of early COVID-19 compliance to and challenges with public health and social prevention measures in the Kingdom of Eswatini, using an online survey

**DOI:** 10.1371/journal.pone.0253954

**Published:** 2021-06-29

**Authors:** Sara Padidar, Shell-may Liao, Siphesihle Magagula, Themb’a A. M. Mahlaba, Nhlanhla M. Nhlabatsi, Stephanie Lukas

**Affiliations:** 1 Department of Biological Sciences, University of Eswatini, Kwaluseni, Eswatini, Swaziland; 2 Department of Physics, University of Eswatini, Kwaluseni, Eswatini, Swaziland; 3 Epidemiology and Disease Control Unit, Ministry of Health, Mbabane, Eswatini, Swaziland; 4 Department of Pharmaceutical and Administrative Sciences, University of Health Sciences and Pharmacy, St. Louis, Missouri, United States of America; Federal University of Rio de Janeiro, BRAZIL

## Abstract

Public health and social measures have been implemented around the world in a bid to prevent the spread of COVID-19. Public compliance with these measures is key in successfully controlling the pandemic. This online survey assessed the compliance and attitude of adults residing in the southern African Kingdom of Eswatini to government protection, activity and travel measures aimed at controlling the spread of COVID-19. A rapid online survey, comprising of 28 questions, was administered in May 2020. More than 90% of respondents knew the virus could kill anyone and most respondents (70%) reported to be compliant to public health and social measures. Females, those who did not use public transport and those aged 30 years and above were significantly (p<0.01) more compliant, particularly to protective and travel measures. Social media, television and official government websites were the primary source of ongoing COVID-19 information for respondents of this online survey, and these methods should continue to be employed to reach the public who regularly use the internet. More than half of essential workers who responded to the online survey reported to have their own personal protective equipment; however, 32% either did not have any protective equipment or shared their equipment with other staff members. Due to the survey being online, these results should not be generalised to populations of low socioeconomic status.

## Introduction

COVID-19 disease, caused by the SARS-COV-2 coronavirus, was identified in 2019 [1]. Coronaviruses are a large family of viruses that cause respiratory infections, which can range from the common cold to more serious diseases. Other coronaviruses that affect humans include Middle East Respiratory Syndrome (MERS) [2] and Severe Acute Respiratory Syndrome [3]. COVID-19 is caused by a new form of coronavirus, first reported in Wuhan (China) in December 2019 [1]. The disease spreads easily from person to person most often through small droplets when a person with COVID-19 coughs, sneezes or speaks and the particles land in the mouth or nose of another and are inhaled into the lungs [4]. Within 2.5 months of being first reported, the virus spread globally and was declared a pandemic [1, 5].

The ideal goal in effectively dealing with a pandemic is to completely stop the spread and eliminate or eradicate the disease [6]. As of the 3^rd^ June 2021, 19 COVID vaccines are in the World Health Organisation Emergency Use List evaluation process [7], with the United Kingdom as the first country to approve and begin mass vaccination on the 8^th^ December 2020 [8]. Ghana and Côte d’Ivoire were the first African countries to begin COVID-19 vaccinations on the 1^st^ March 2021 [9]. However, during the first 11 months of the pandemic, there were no commercially available vaccine or specific antiviral treatment for COVID-19 [10] and The World Health Organisation advised the best way to prevent the transmission of COVID-19 was by avoiding exposure to the virus [11]. In response to the pandemic, almost all countries have implemented public health and social measures [12]. These measures aim to slow the transmission of COVID-19. Slowing the spread of a pandemic reduces the number of active cases at a given time, known as ‘flattening the pandemic curve”. This allows the health system (doctors, nurses, hospitals) time to prepare and respond without being overwhelmed [13]. Adherence to public health and social mitigative measures are therefore key to flattening of the pandemic curve.

Studies investigating compliance of public health and social measures to prevent the spread of COVID-19 have so far shown a spectrum of compliance to such measures [14–22]. Reasons for compliance, or lack thereof, to prevention measures have included attitudes to the prevention measures, perceived risk of the virus to the individual, belief in the existence of the virus, law enforcement and / or penalties imposed due to non-compliance, availability and ease of access to protective measures (e.g., face masks, running water and soap, hand sanitiser), and availability and accessibility of alternative work and travel arrangements [14–22].

Examples of measures include:

- Personal protective measures–e.g., wearing of masks, washing or sanitising of hands frequently, practicing respiratory etiquette
- Social measures–e.g., staying and working at home, staying at least one meter away from another person not from your household, and avoiding crowded settings (e.g., public transport, bars, and schools)
- Travel measures–e.g., limiting travel between cities, regions, and nations.

These limitations–especially in relation to limited travel, interactions, and reduced access to work–have led to a global emergency that is not only a health crisis but also an economic and social crisis [23]. Public health and social measures have seen numerous workers having either lost their jobs or working on reduced schedules [24]. In low- and middle-income countries, few nations have the resources and infrastructure to provide welfare relief to citizens to mitigate for the loss of income [25]. In the Kingdom of Eswatini, 30% of the population live under $1.90 a day [26], and the country is braced for a 25% reduction in income through the Southern African Customs Union in 2022 leading to both short- and medium-term economic effects of the pandemic [27]. Lack of government support and little or no relief systems in place, employees may need to leave home and travel by public transport to work. In southern Africa, including Eswatini, public transport systems usually comprise of overcrowded licensed minibuses. In Eswatini, such transport is used by up to 90% of the population for daily travel, including across borders [28]. Overcrowded public transport have been associated with the transmission and acquisition of airborne diseases including tuberculosis and influenza [29, 30]. For this reason, many countries, including Eswatini, shut down, staggered timings or decreased occupancy on public transport systems [31–33].

To reduce the spread of the virus, many governments around the world advised the public to stay at home [12]. However, epidemic models have shown airborne infectious diseases are more easily spread within households, and contact density is the main driver in epidemic spread [34]. In Eswatini, average household size is four people and over 40% of households have 3 or more persons per sleeping room [35].

### Status of COVID-19 pandemic in Eswatini (formerly known as Swaziland)

On the 14^th^ March 2020, the southern African, landlocked Kingdom of Eswatini announced its first case of COVID-19. With eight confirmed cases, on the 27th March 2020, Eswatini introduced public health and social measures in the form of a partial country lockdown on selected sectors of the economy to curtail the spread of the SARS-COV-2 virus [36]. Essential sectors of the economy (such as health, agriculture and telecommunications) were permitted to operate under strict hygiene conditions, whilst other businesses such as carwashes, furniture shops, salons and bars were closed [37, 38]. Public transport operators were allowed to operate at 70% capacity with all passengers required to wear a mask [32].

One month into the partial lock down, with 1 death and 32 cases (97% from Hhohho and Manzini regions mainly in men aged 18–44 years of age) [39], the Government announced there had been public complacency to social distancing measures and other measures to control the spread of the disease [40]. Reliance on public transport and the global shortage of personal protective equipment due to the rapid high demand for the products in response to the pandemic [41] further compounded chances of spreading of the disease to and by frontline essential workers [42]. Tackling the spread of COVID-19, much like other infectious diseases, requires a coordinated effort by both the health system–needing the infrastructure, commodities and capacity to deal with the disease–and the public–requiring behaviour change.

To this end, we carried out a rapid online survey to better understand the Eswatini public’s compliance to government measures taken to curtail the spread of the SARS-COV-2. We also aimed to determine challenges to adherence by the public to the prevention of the spread of COVID-19, any symptoms experienced, as well as their beliefs and sources of information about COVID-19. Data from this rapid online survey was used by the Ministry of Health to inform better-targeted public health interventions and education messaging to reach target populations during the evolving pandemic crisis. During the time of the survey, confirmed COVID-19 cases in Eswatini had risen from 187 to 285, with over 70% of cases from Hhohho and Manzini regions, 52% of cases in males, 59% of cases asymptomatic, and 18–44 year olds accounting for 70% of all SARS-COV-2 positive confirmed cases [39].

## Methodology

### Ethical approval

Necessary IRB and ethical committee approvals were obtained from Ministry of Health (Kingdom of Eswatini) and University of Health Sciences and Pharmacy in St Louis (USA). The survey was registered with the Eswatini National Health Research Review Board. The survey started with a consent statement and participants who gave consent to willingly participate in the survey would click the ‘Continue’ button and be directed to complete the self-administered questionnaire. Respondents were free to terminate the survey at any time and no identifying information was captured.

### Study design and instrument

A cross-sectional anonymous voluntary online survey was administered between 14^th^ May to 31^st^ May 2020, during the COVID-19 pandemic emergency. SurveyMonkey, a secure, password protected platform, was used to host the survey [43]. To keep the survey anonymous, no identifiable information was requested from participants or captured using the SurveyMonkey platform. For added security, survey data was downloaded from the SurveyMonkey platform and stored on a password secured database for analysis. All data pertaining to the survey was deleted from SurveyMonkey platform after download.

The online survey consisted of 28 multiple-choice questions and these are detailed in S1 Text. Questions covered four broad areas: 1) sociodemographic characteristics, 2) compliance to public health and social measures in preventing the spread of COVID-19, 3) challenges to compliance to prevention measures (e.g., overcrowding, access to personal protective equipment for essential workers, being a contact of a COVID-19 case, space on public transport) and 4) beliefs and sources of information for COVID-19. The survey asked participants about COVID-19 symptoms they or their household may have experienced. Questions were reviewed, validated and refined by independent experts for appropriateness and relevance. Each of the compliance questions were written to mirror the Eswatini Government’s public health and social measures [40, 44]. The survey was available in English and siSwati, and participants had the choice to complete the survey in either language.

To eliminate any discrepancies and biases that would have been introduced with a change in policy, the stopping rule was a governmental change to the required measures. The online survey was administered between 14–31 May, inclusive. During this time, there were no changes to the public and social measures implemented by the Government. On 29^th^ May 2020, the Government announced relaxation of some of the public health and social measures–effective from the 1^st^ June 2020 [45]. Consequently, the survey was closed on the 31^st^ May 2020.

During the survey period, the investigators accessed the data twice (after two days and after one week of launching the survey) to assess data quality. This did not affect participants accessing the survey nor affected the survey recruitment process.

### Variables studied

Sociodemographic variables studied in this survey included gender, age, region of residence, education level, subscription to private medical insurance, being an essential worker, household density (measured by number of people in a household and the number of rooms in a house) and use of public transport. Ages were grouped in years as 18–29, 30–44, 45–59 and 60 years and over. This grouping was used as studies had shown 18–44 year olds to be the main internet users in Eswatini [46]. Essential workers were asked which of the government’s essential sectors [36–38] they were employed.

### Target audience and recruitment process

The target audience for this study were adult residents of Eswatini who used the internet. Only respondents who identified themselves as living in one of the four regions of Eswatini were included in the analysis. Those who indicated they lived outside of Eswatini or did not indicate living in any region of Eswatini were excluded in the analysis. The survey only allowed respondents to access the survey once through any one device or browser. Additionally, respondents were asked if they had already completed the survey. Respondents who indicated they had previously completed the survey were excluded in the analysis. Other than the exclusions discussed above, all attempted survey responses were included regardless of how many questions were answered.

In Eswatini, approximately half of the population use the internet (542,400 users), and a quarter of the population actively use social media with Facebook being the most popular (250,000 users per month), and Twitter the second most (20,000 users per month) [46]. Therefore, when calculating the minimum sample size (n = 384), a population size of 542,400 at the 95% confidence interval with a 5% margin of error was used [47]. Mobile phones are used to access the internet by 97% of social media users, and 99% of the population has a mobile phone connection although affordability of both devices and services varies [46]. Consequently, a broad, multi-pronged recruitment campaign was implemented to reach as many internet users as possible:

- To capture the public who are following COVID-19 developments in Eswatini but are not necessarily social media users:
  ○ An invitation to the survey was embedded on the Eswatini COVID-19 dashboard website (mirrored on Ministry on Health and the national university (University of Eswatini) websites).
  ○ A pop-up invitation to the survey was embedded on the University of Eswatini’s COVID-19 website, a platform established for both the academic community and the general public to learn about COVID-19, inviting all visitors to the site to the survey.
- To capture the public who are social media users and not necessarily following COVID-19 developments in Eswatini:
  ○ An invitation to the survey was sent out using social media platforms such as Twitter and Facebook.
- To capture the public who may or may not be following COVID-19 developments in Eswatini and may or may not be social media users:
  ○ At the end of the survey respondents were invited to forward the survey invitation and link to their contacts e.g., friends and family.
  ○ National radio, TV and print media outlets reported the existence of the study to the public.

Survey invitations were written in English and in siSwati. The online survey was voluntary, and respondents were not compensated for their participation.

### Compliance scoring

Five of the 28 multiple-choice questions were used to calculate overall compliance in terms of protection, activity, and travel (Table 1). Each component of overall compliance was weighted equally i.e., maximum score of 30 points for protection, activity and travel respectively. The higher the score, the more compliant the respondent was to government public and social measures [40, 44, 48]. The scoring system, described below allowed the data to be quantified for statistical analysis.

**Table 1:** Survey questions and scored answers related to compliance.

Survey question	Choice of answers	Assessment points
**Compliance–personal protection (max 30 points): If more than one ticked, points added for each precaution taken as more compliant**		
What precautions have you been taking to protect yourself against COVID-19 (tick all that apply)?	Washing or sanitizing hands	7.5 points
	Social distancing (deliberately keeping more than 1m away between you and someone else who does not live with you)	points
	Staying home	7.5 points
	Wearing a mask at all times when away from your home	7.5 points
	None of the above	3 points
Compliance–activity* **(max 30 points): If more than one ticked, lowest score given as less compliant**		
What are the reasons for leaving your home (tick all that apply)?	Work	30 points
	Shopping	30 points
	Medical reasons	30 points
	Visiting other homes	15 points
	Other (please specify)	3–30 points**
**Compliance–travel (max 30 points): Each of the three questions summed for a total travel score**		
During the past seven days, how many times did you leave your home?	Never	10 points
	Once	8 points
	1–2 times	6 points
	3–5 times	3 points
	6 or more times	1 point
In the last one month, have you travelled to your village homestead?	Yes, several times (e.g., 3 times or more)	1 point
	Yes, once or twice	5 points
	I live at my village homestead	10 points
	No, I do not have a village homestead	10 points
	No, I stayed at home	10 points
Over the Easter holidays or recent public holidays, did you travel within the country to visit friends or family?	Yes	1 point
	No	10 points

*In the survey, this question came straight after the question “During the past seven days, how many times did you leave your home?” (see S1 Text).

**Scores given for essential (30), semi-essential (15) and non-essential (3) as described by the Government of the Kingdom of Eswatini [40, 44, 48].

#### Personal protection

An additive score was calculated by adding each protective measure outlined by the Government of Eswatini [40, 44, 48] (e.g., wearing facemask at all times when away from home, washing or sanitizing hands, staying home, social distancing) that the respondent indicated to be taking. Each protective measure was weighted equally, and the greater the number of protective measures, the greater their score, with a 30-point maximum.

#### Activity score

Respondents were asked their reason(s) for travelling outside of the home. Activities were categorised as essential (e.g., seeking medical attention, shopping), semi-essential (activities that could be done from home but were not banned e.g., attending church, studying) and non-essential (e.g., socialising, visiting friends and family) as described by the Government of the Kingdom of Eswatini [40, 44, 48]. Each category received a score (essential = highest score (more compliant), non-essential = lowest score). Where individuals reported to carry out two or more types of activities (e.g., essential and a semi-essential, essential and non-essential, semi-essential and non-essential) activities, the score for the least essential (less compliant) activity was used to compute the activity score. The rationale for this approach was twofold, 1) individuals who carried out less essential activities were not complaint to the government’s public health and social measures and were thus putting themselves and others at greater risk of contracting COVID-19 by engaging in such activities, and 2) the survey did not ask the frequency each activity was carried therefore a weighted score could not be reasonably calculated.

#### Travel score

A composite score was calculated based on respondent’s frequency of travel during the week, during public holidays and to village homesteads (Table 1), with a total 30-point maximum. The more frequent the person travelled, the lower their score because they were less compliant [44]. Essential workers were awarded the maximum score (more compliant) for their weekly travel frequency when their reason for travel was related to essential activities only (e.g., if an essential worker left their house six or more times, but their reason for leaving was only work, they would be considered compliant and receive the maximum score of 10 points for weekly travel).

#### Overall compliance percentage

Protection, activity, travel scores were equally weighted and used to determine an overall compliance percentage score.

### Challenges, beliefs and information

Survey questions also examined the challenges of complying with public health and social measures as well as COVID-related beliefs and information sources. To analyse the challenges faced by participants, survey questions probed on the availability of personal protective equipment for essential workers, contact with a confirmed COVID-19 positive person, overcrowding in the home, and space on public transport based on the current recommendations in place while the survey was open [11, 32, 41]. Two questions were included to better understand where participants first learned about COVID-19 and what sources they use to stay up to date. An additional question on what participants believe COVID-19 to be was added to explore links between beliefs, information sources and compliance.

### Data analysis

Responses from the survey were tabulated, translated into English when applicable, and scored for compliance as described above. Descriptive statistics were calculated for all independent variables and focussed on frequencies and percentages. As the data was not normally distributed, Kruskal-Wallis test [49] with a paired Dunns’ multiple comparison test using the Bonferroni method [50, 51] was used to determine the significance between sociodemographic variables and overall compliance or a particular compliance category.

Stepwise backward elimination generalised linear models with gamma distribution was conducted to identify associations between independent variables (gender, age, region, education, medical aid, public transport user, essential worker, number of people in household, number of rooms in house) that best explained overall compliance [52]. At each step, ANOVA was used to compare the fits of each model [53].

Chi-squared test of independence was used to measure association between categorical variables. The Cramér’s statistics (Cramér’s V) was used to interpret these association estimates and this method is often reported in addition to chi-squared tests as an effect size index [54, 55]. Cramér’s V index ranges from 0 to +1 and a higher Cramér’s V value indicates a stronger association between variables, whilst a lower one indicates a weaker association [55].

A sensitivity analysis (multinomial logistic regression and predictive mean matching) was carried out for the surveys without answers to the compliance questions. The sensitivity analysis is performed to explore the result of the analysis under alternative scenarios for the missing data. This is done to predict both the direction and the magnitude of the missing data had it been observed. Up to 16 questions did not receive an answer by at least one respondent. These questions were mainly towards the end of the survey and belonged to up to 15% of respondents who had answered less than half of the survey. The missingness of the data was categorised as item non-response data. Mice imputation method was used to replace for missing data items [56].

Statistical significance was determined by p values <0.05. All statistical analyses were carried out in ‘R’ version 3.5.1 [57], using libraries gmodels [58], AICcmodavg [59], lme4 [60], SparseM [61], naniar [62], mice [63], and FSA [64].

## Results

### Overview

This is the first online survey to measure public compliance, perceptions and challenges to compliance to public health and social measures against COVID-19 in Eswatini. A total of 488 respondents completed the survey. Of these, 21 were excluded as respondents identified themselves as living outside of Eswatini or did not answer this question. Eleven respondents indicated they had completed the survey before (with one respondent indicating they had both previously completed the survey and was living outside of Eswatini). Therefore, data from a total of 457 respondents was analysed.

Results from the survey covered four broad areas: 1) sociodemographic characteristics, 2) compliance to public health and social measures in preventing the spread of COVID-19, 3) challenges to compliance with prevention measures, and 4) beliefs and sources of information for COVID-19.

#### 1) Sociodemographic characteristics

There was no significant difference in the number of females (52%) and males (48%) that participated in the survey, and this is reflective of the population of Eswatini [35]. Most respondents (83%) were between the ages of 18–44 years (Table 2), reflecting the age group that typically uses the internet in Eswatini [46]. Respondents lived in all four regions of Eswatini, with most respondents coming from Hhohho (39%) and Manzini (41%) regions. These two regions are also the most populous regions in Eswatini [65].

**Table 2:** Sociodemographic data of survey respondents.

Variable	No. of participants in survey (%)	2017 Eswatini census data [65]±	Overall compliance percentage score			
			Mean ± SD	Kruskal-Wallis Test		
				χ^2^	df	p-value
**Gender**				18.992	1	**<0.001**
Males	220 (48.1)	272909 (47.1)	68 ± 32			
Females	237 (51.9)	306974 (52.9)	77 ± 29			
**Age**				10.821	3	**0.013**
18–29 years	201 (44)	203255 (35.1)	67 ± 34			
30–44 years	179 (39.2)	203047 (35.0)	75 ± 31			
45–59 years	66 (14.4)	101912 (17.6)	82 ± 19			
60+ years	10 (2.2)	71669 (12.4)	81 ± 30			
Did not answer	1 (0.2)		80 ± 0			
**Region**				10.364	3	**0.016**
Hhohho	177 (38.7)	286534 (29.4)	78 ± 26			
Lubombo	49 (10.7)	187931 (19.3)	64 ± 34			
Manzini	186 (40.7)	319527 (32.7)	73 ± 32			
Shiselweni	45 (9.8)	181765 (18.6)	63 ± 38			
**Medical aid**				15.253	1	**<0.001**
Yes	189 (41.4)	N/A	79 ± 27			
No	268 (58.6)		68 ± 33			
**Education**				11.707	6	0.069
Some primary	1 (0.2)		50 ± N/A			
Primary completed	2 (0.4)	(30.0)	63 ± 42			
Some high school	4 (8.8)		50 ± 41			
High school completed	62 (13.6)	(39.2)	70 ± 32			
Some university	84 (18.4)		67 ± 34			
University completed	209 (45.7)	(7.2)	73 ± 31			
Post-graduate	94 (20.6)		80 ± 25			
Did not answer	1 (0.2)		73 ± 0			
**Essential worker**				4.377	1	**0.036**
Yes	129 (28.2)	N/A	74 ± 33			
No	328 (71.8)		72 ± 30			
No. of people in household		4.02[35]		4.117	3	0.249
1	52 (9.8)		62 ± 41			
2–3	124 (27.1)		64 ± 37			
4–5	155 (33.9)		70 ± 35			
6 or more	126 (27.6)		63 ± 35			
No. of rooms in house**		2.84 [35]		7.704	3	0.053
0–1	45 (9.8)		55 ± 40			
2–3	183 (40.0)		63 ± 37			
4–5	153 (33.5)		68 ± 36			
6 or more	75 (16.4)		73 ± 31			
Did not answer	1 (0.2)		25 ± 0			
**Public transport user**				12.853	1	**<0.001**
Yes	102 (22.3)		76 ± 16			
No	302 (66.1)	N/A	84 ± 21			
Did not answer	53 (11.6)		1.8 ± 13			

*excludes kitchen and bathroom, SD = standard deviation, χ^2^ = chi-square, df = degrees of freedom. P values of significant variables highlighted in **bold**.

± Census data: Gender and age data reported as ages 20 years and older; region data reported is for the entire population (including those under 18 years of age); education data reported as a highest level of education attended for ages 15–35 years and only reported as a percentage at primary, secondary and tertiary levels.

More than 60% of participants had completed tertiary education, 66% of respondents did not use public transport and 41% belonged to a private medical insurance scheme (Table 2). This is not representative of the population of Eswatini [35, 65]; however, this is representative of the socioeconomic background of the target population (internet users) due to the high costs involved both in purchasing devices and data to access the internet in Eswatini.

#### 2) Compliance of individuals to precautionary measures against COVID-19

Respondents were asked five questions regarding their personal compliance to public health and social measures against COVID-19, which were categorised across protection, activity and travel (Table 1). Using stepwise backward elimination generalised linear modelling starting with the full model (containing all variables) the best performing model included gender, age, education, medical aid, public transport users and essential workers (Table 3). The parameters that were significantly associated with overall compliance in this model are males, essential workers, and those who use public transport (Table 3).

**Table 3:** Summary of parameter estimates (β), standard error (SE), confidence intervals (CI) and p value for the best performing model.

	β	SE	p-value	2.5% CI	97.5% CI
*Intercept*	1.140e-02	5.328e-04	< 2e-16	0.0104	0.0125
Age:					
30–44	-2.407e-04	3.371e-04	0.476	-0.0009	0.0004
45–59	-2.826e-04	4.243e-04	0.506	-0.0011	0.0005
60+	-1.127e-03	8.006e-04	0.114	-0.0028	0.0036
**Gender: males**	**1.014e-03**	**2.541e-04**	**<0.0001**	**0.0005**	**0.0015**
Medical aid: yes	-2.888e-04	2.872e-04	0.315	-0.0009	0.0003
No. of people in household:					
2–3	1.462e-04	4.267e-04	0.732	-0.0007	0.0009
4–5	6.194e-04	4.097e-04	0.131	-0.0002	0.0014
6+	7.865e-04	4.366e-04	0.072	-0.0001	0.0016
Education:					
< primary	7.637e-03	4.077e-03	0.062	0.0006	0.0167
Primary	4.032e-03	2.339e-03	0.086	-0.0017	0.0090
Some high school	1.544e-04	1.812e-03	0.392	-0.0017	0.0054
Some university	-1.422e-04	4.591 e-04	0.757	-0.0010	0.0008
University	-4.087e-04	4.230e-04	0.923	-0.0008	0.0009
Post-graduate	-5.880e-05	4.8749e-04	0.904	-0.0009	0.0010
**Essential worker**	**-6.415e-04**	**2.953e-04**	**0.030**	**-0.0012**	**-0.0006**
**Public transport user**	**9.619e-04**	**3.132e-04**	**0.002**	**0.0003**	**0.0016**

Significant parameters are indicated in **bold**.

**Fig 1:**
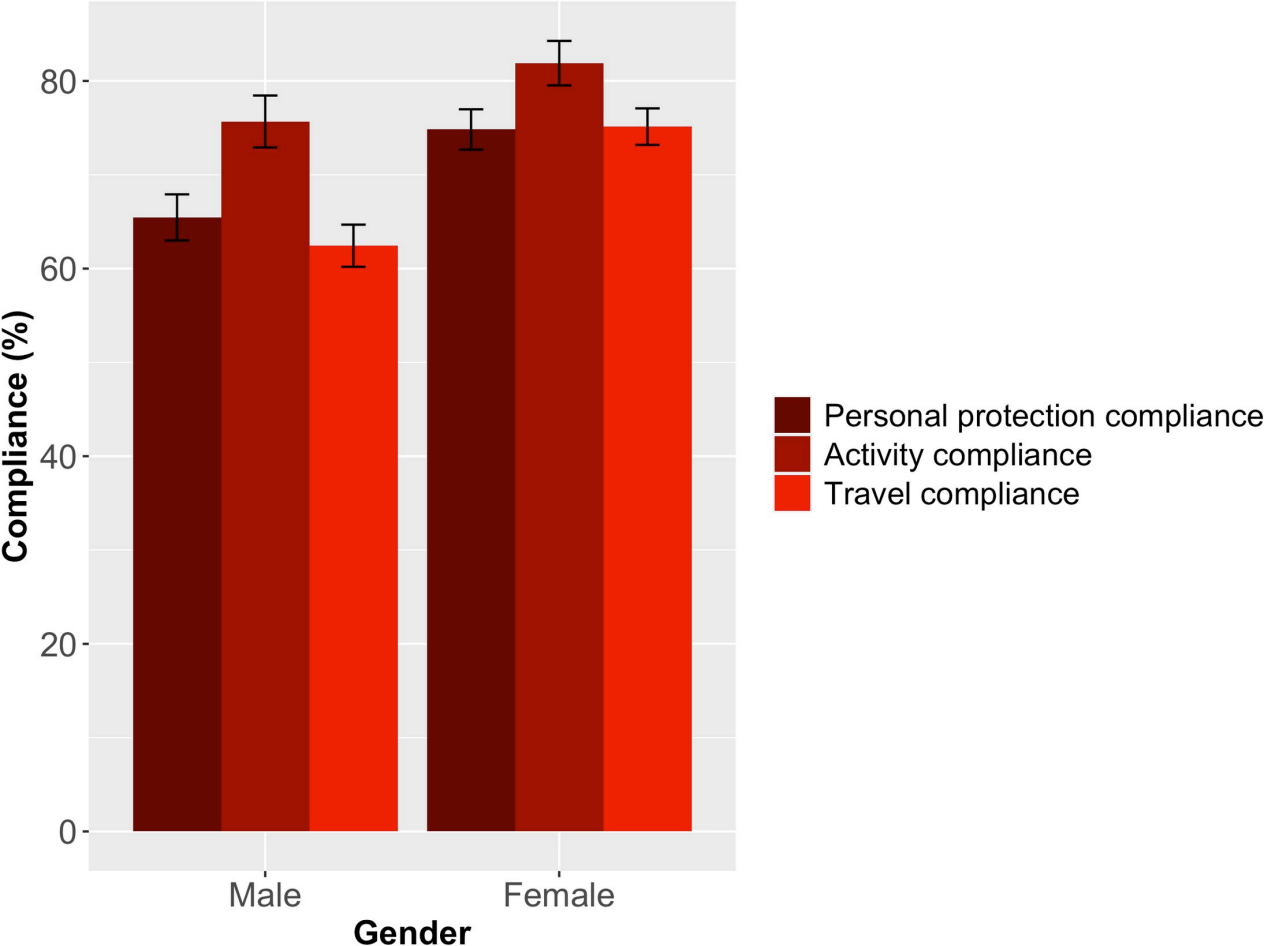
Mean compliance for each compliance component by gender (Protection measures: KW χ^2^ = 8.327, d.f. = 1, p<0.001, Activity: KW χ^2^ = 2.665, d.f. = 1, p>0.05, Travel measures: KW χ^2^ = 23.285, d.f. = 1, p<0.001).

Gender was a highly significant variable for overall compliance (Kruskal Wallis (KW) χ^2^ = 18.992, d.f. = 1, p<0.001, Table 2) with females more compliant than males. The post-hoc Dunn’s multiple comparison with Bonferroni adjustment showed significantly more females than males reported to be compliant in all aspects of protecting themselves from COVID-19 (e.g., wearing a mask when out of the home, washing or sanitising hands, staying at home, and being socially distant) (p<0.001), and with all government travel advice (p<0.001), as shown in Fig 1 (also see S1B–S1E Fig). Approximately 10% more women (compared to men) reported washing or sanitising their hands, social distancing, staying at home, and wearing mask (S1B–S1E Fig). Almost 60% of women had travelled between 0–2 times in the last 7 days compared with 37% of males. Most women (80%) did not report travelling to visit friends and family over the public holidays, compared with 67% of males. Over 70% of women reported to have not travel to their village homestead, did not have one or lived there, compared with 54% of males (S1B–S1E Fig). There was no significant difference (p>0.05) between males and females for compliance in the type of activities carried out whilst away from home (e.g., essential activities such as work, shopping, seeking medical treatment), Fig 1.

**Fig 2:**
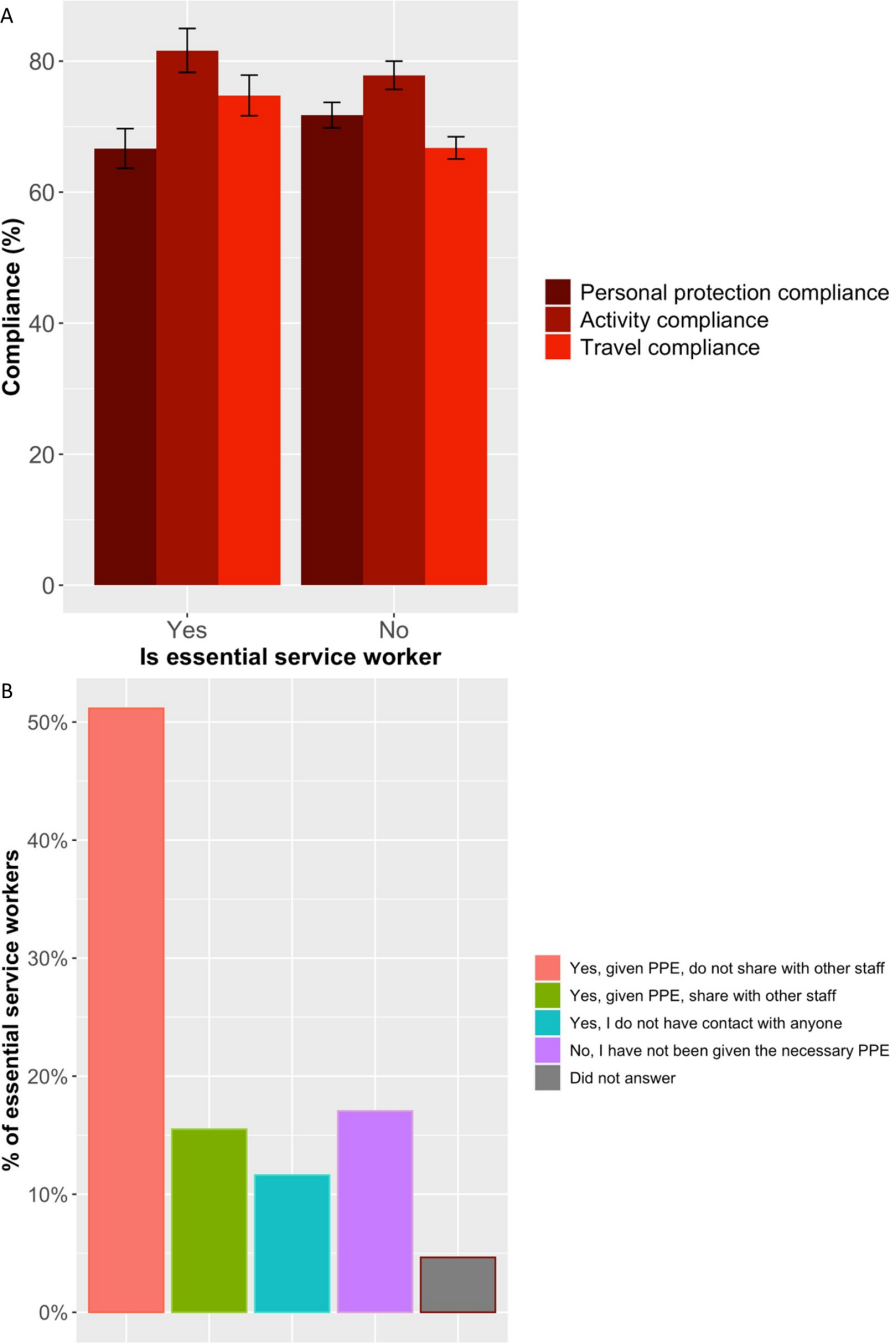
A. Mean compliance for each compliance component by essential worker. B. Personal Protective Equipment (PPE) for essential workers, Yes = essential workers. No = not essential workers.

A total of 129 essential workers responded to the survey, representing 13 areas of employment (S2C–S2G Fig). There was a significant positive association between being an essential worker and overall level of compliance to prevention measures according to the chi-square test of independence (χ^2^ = 21.287, d.f. = 3, p<0.01), with a weak effect size (Cramer’s V = 0.18). It is interesting to note, average overall compliance to public health and social measures was reported to be lowest in the food and agriculture sector at 81.3%, network infrastructure (81.5%) and IT systems and telecommunication sectors (85.4%) (S2C–S2G Fig). The post-hoc Dunn’s multiple comparison with Bonferroni adjustment showed significantly more essential workers reported to be compliant in all aspects of protecting themselves from COVID-19 except staying at home (p<0.05) where only 35% of essential workers reported staying at home compared with over 70% of respondents who were not essential workers, as shown in Fig 2A and S2C–S2G Fig. Significantly more essential workers were compliant with government travel advice (p<0.001) (Fig 2A). When this data was disaggregated, 56% of essential workers and 65% of non-essential workers had not travelled to their village homesteads in the last month, there were no differences between essential and non-essential workers with regards to travel over the public holidays, and 20% of essential workers and 60% of non-essential workers had left their homes 0–2 times in the past 7 days (S2C–S2G Fig). Note, the scoring methodology (Table 1) did not penalise essential workers for travelling frequently for work and essential purposes. There was no significant difference (p>0.05) between essential and non-essential workers for compliance in the type of activities carried out whilst away from home (e.g., essential activities such as work, shopping, seeking medical treatment), S2C–S2G Fig.

**Fig 3:**
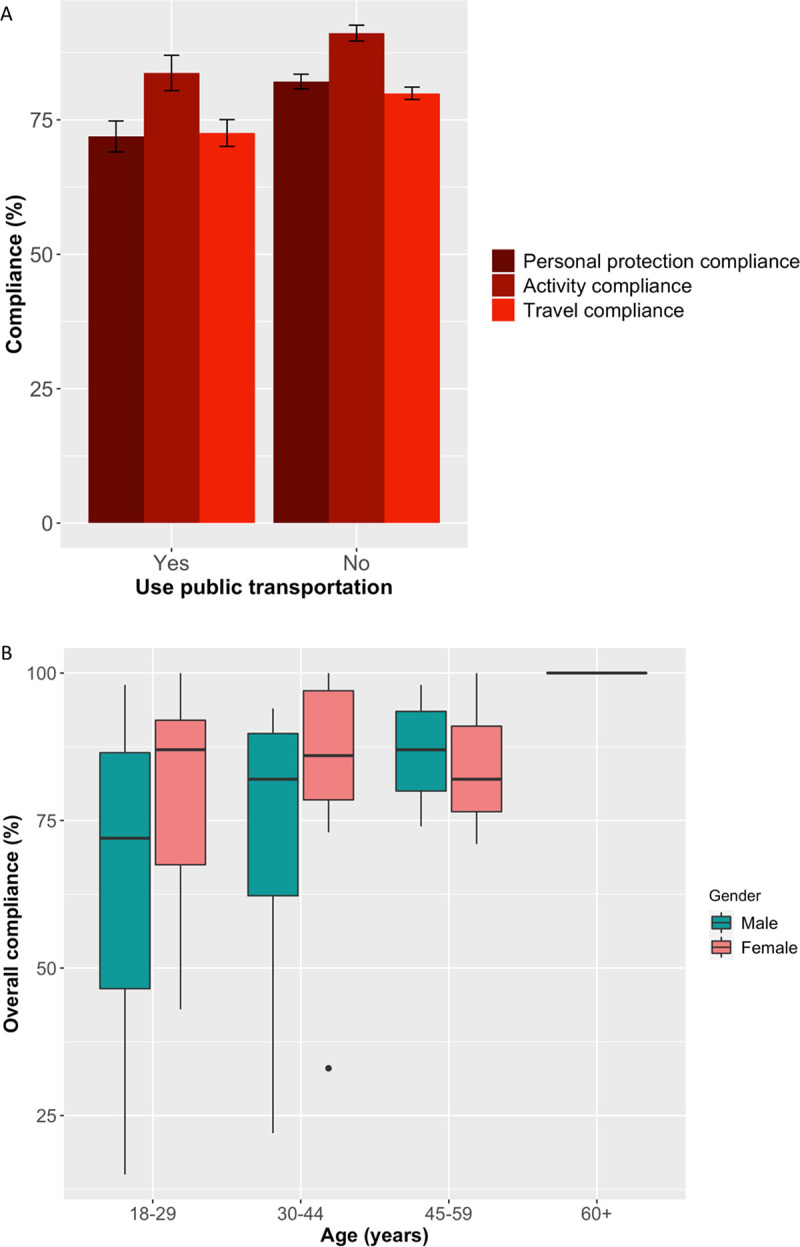
A. Mean compliance for each compliance component by public transport user. B. Overall compliance score of public transport users, by gender and age.

There was a significant difference (KW χ^2^ = 12.853, d.f. = 1, p<0.01, Table 2) between those who did and did not use public transport with overall compliance to government public health and social measures, with public transport users significantly less compliant to government measures. There were 53 respondents who did not identify if they used public transport. These 53 respondents also did not answer any of the compliance questions. The post-hoc Dunn’s multiple comparison with Bonferroni adjustment showed significantly fewer public transport users reported to be compliant in all aspects of protecting themselves from COVID-19 (except staying at home, p<0.01), type of activities carried out whilst away from home (p<0.05) and with all government travel advice (p<0.05), as shown in Fig 3A (also see S3C–S3F Fig). A third of public transport users reported visiting their village home in the last month and a quarter had left their home 6 or more times in the past week (S3C–S3F Fig). Female public transport users were significantly more complaint overall than males (KW χ^2^ = 23.285, d.f. = 1, p < 0.01); however, age was not a significant variable in overall compliance for public transport users to overall public health and social measures (Fig 3B). The chi-square test of independence showed there was no significant association between public transport users and essential worker (χ^2^ = 1.348, d.f. = 1, p>0.05), indicating the essential workers that responded to this online survey were just as likely to not use public transport as they were to use public transport.

**Fig 4:**
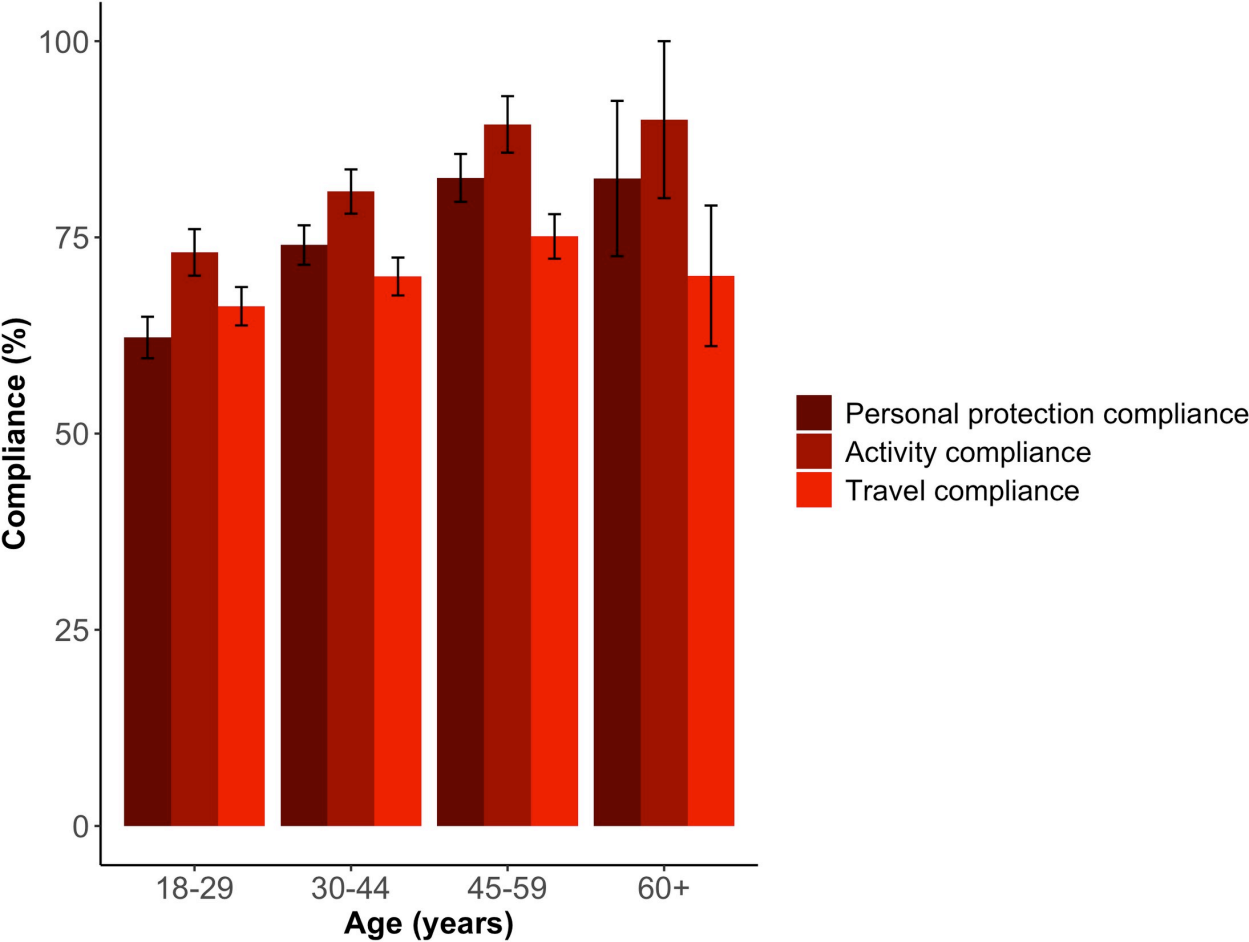
Mean compliance for each component by age.

The Kruskal-Wallis test also showed that overall compliance significantly increased with age (χ^2^ = 10.821, d.f. = 3, p<0.05, Table 2), with 18–29 year olds least compliant for personal protection compared to 30–44 year olds (p<0.001) and 45–59 year olds (p<0.01), according to the Dunn’s multiple comparison test. Across the personal protection categories, 18–29 year olds were less likely to practice social distancing, whilst the other age groups were less likely to practice staying at home (Fig 4 and S4B–S4E Fig). The Dunn’s multiple comparison test also showed that respondents aged 18–29 years were also significantly less compliant than 45–59 year olds for the types of activities carried out whilst away from home (p<0.05). There was no significant difference (KW χ^2^ = 1.502, d.f. = 3, p>0.05) between ages for travel compliance (Fig 4, also see S4B–S4E Fig). The 60 or older age group comprised of 10 respondents, which was too small to determine if there were any significant differences.

Additionally, the Kruskal-Wallis test revealed significant differences between the overall compliance between the regions (χ^2^ = 10.634, d.f. = 3, p<0.05. Table 2). The pairwise post-hoc Dunn test (p<0.05) showed residents of Hhohho region to report being significantly more compliant overall than those living in Lubombo region. It is not clear why this regional difference was observed and this variable was removed in the generalised linear model (Table 3); however, five months after the survey ended and public health and social measures relaxed, confirmed cases of COVID-19 from Hhohho region have remained significantly greater than Lubombo region [39]. This is likely to be due to the greater population density in Hhohho region and/or socioeconomic differences between the two regions affecting accessibility to an online survey.

Finally, there were no significant differences in overall compliance between education levels, number of people living in a household and the number of rooms in the house (Table 2). Of these non-significant variables, only education and the number of people living in a household remained in the best performing model (Table 3).

#### 3) Challenges to complying with public health and social measures

Public health and social measures aim to minimise risk of the public acquiring COVID-19. However, challenges may remain outside the individual’s immediate control.

*Personal protective equipment*. Most essential worker respondents worked in the health sector as well as the food and agriculture sector, which is representative of the employment in Eswatini. No respondents self-identified to be an essential worker from the public transport sector (S2C–S2G Fig). More than half of essential workers said they felt protected from COVID-19 at work because they had the necessary Personal Protective Equipment (PPE) which was not shared with other staff members, 11.6% of essential workers reported feeling protected because they did not meet anyone at work; however, 15.5% of essential workers shared their PPE and more than 17% did not have the necessary PPE (Fig 2B). The survey did not ask respondents about the type of PPE they had or shared.

*Contact with a confirmed COVID-19 positive person*. Only 3% of respondents said they were contacts of a confirmed COVID-19 case, whilst 54% said they were not, 31% were not sure and 12% did not answer the question. Respondents were also asked if they were screened or tested for COVID-19. Less than a quarter of respondents had been screened for COVID-19, with 2% of those screened / tested were identified to be positive for COVID-19 and a further 4% still awaiting results. The chi-square test of independence showed that there was no significant association between those that identified to be contacts of a confirmed COVID-19 case and overall compliance (χ^2^ = 5.5595, d.f. = 6, p>0.05). There was a highly significant positive association (χ^2^ = 19.845, d.f. = 2, p<0.001) between those who had been tested and those that knew they were contacts of COVID-19 confirmed cases, indicating government screening-testing-contact tracing measures were being carried out. The effect size (Cramér’s V) was found to be moderate (0.22). Tracing measures have been recommended for COVID-19 by WHO [66] and for other diseases in Eswatini such as TB [67].

Most respondents (70%) did not have any symptoms of COVID-19. Of the respondents who stated they had symptoms, 74% had only one symptom, 16% reported to have two symptoms, 5% had three symptoms, 1% had four symptoms, and 4% had all five symptoms of COVID-19. These were the symptoms of COVID-19 that were known at the time of the survey. New symptoms have since been identified (e.g., loss of smell). According to the chi-square test of independence, there was no significant association between having at least one symptom and overall compliance (χ^2^ = 2.3818, d.f. = 3, p>0.05), however, being in contact with a confirmed case of COVID-19 was significantly positively correlated with the following symptoms experienced by respondents during the last two weeks: dry cough (χ^2^ = 9.332, d.f. = 2, p<0.01), shortness of breath (χ^2^ = 7.272, d.f. = 2, p<0.05), flu-like symptoms (χ^2^ = 15.200, d.f. = 2, p<0.001) and no symptoms (χ^2^ = 11.362, d.f. = 2, p<0.01). However, the effect sizes were all found to be weak (0.13–0.19).

**Fig 5:**
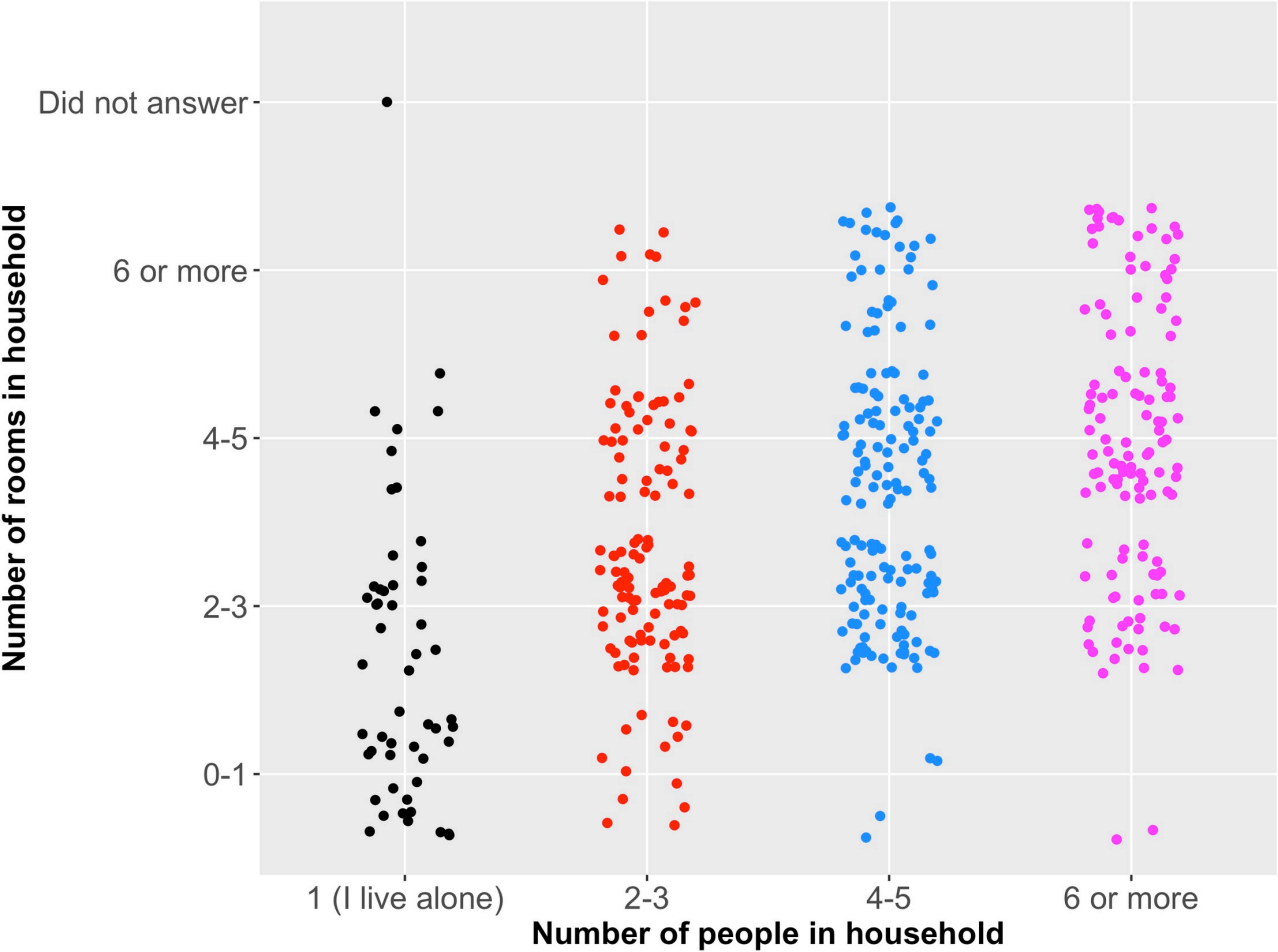
Association between household size and number of rooms in house.

*Overcrowding in the home*. Most respondents (34%) said they lived in a household comprising of 4–5 people, with 40% of respondents living in a house with 2–3 rooms (excluding kitchen and bathroom) (Table 2). The chi-square test of independence revealed that there was a significant positive association (χ^2^ = 144.840, d.f. = 9, p<0.001) with household size and number of rooms in the home. Nineteen (4%) of respondents would not be able to isolate a member of the family in a dedicated room as they lived with others in a one-room house (Fig 5).

Most respondents (71%) reported household members complied with government guidance on personal protective measures (e.g., wearing a mask when outside of the home, social distancing etc., see question 21 in S1 Text). There was no significant difference between household size and household’s compliance to protective measures (KW χ^2^ = 4.117, d.f. = 3, p>0.05) (Table 2). There was also no significant difference between the number of rooms in a house and the household’s compliance to protective measures (KW χ^2^ = 7.704, d.f. = 3, p>0.05) (Table 2). Using the chi-square test of independence, there was a significant positive association (χ^2^(121) = 15723, p<0.001) between symptoms of respondents and those of their household, with shortness of breath being most strongly associated (Cramer’s V = 0.72).

*Space on public transport*. Public transport users were asked if there was space for at least one person between them and the next passenger, as per government guidance [32]. Most respondents (84%) said government advice was being adhered to on public transport.

#### 4) Beliefs and sources of information for COVID-19

The survey asked participants what they believed COVID-19 to be (question 14 in S1 Text). More than 90% of respondents believed COVID-19 can kill anyone; however, 6% of respondents declined to answer this question. Two percent of respondents believed COVID-19 did not exist. A total of eight respondents (2%) believed COVID-19 “kills only Americans, Chinese and Europeans”. According to the chi-square test of independence, there was no significant association between education level, gender or age with respondents’ understanding of COVID-19 (χ^2^ = 76.203, d.f. = 12, p>0.05; χ^2^ = 1.653, d.f. = 2, p>0.05; and χ^2^ = 14.696, d.f. = 6, p>0.05, respectively). Additionally, there was no significant association between beliefs and overall compliance to public health and social measures (χ^2^ = 7.875, d.f. = 9, p>0.05), but this could be due to the small number of respondents (n = 15) who did not believe the COVID-19 could kill anyone.

**Fig 6:**
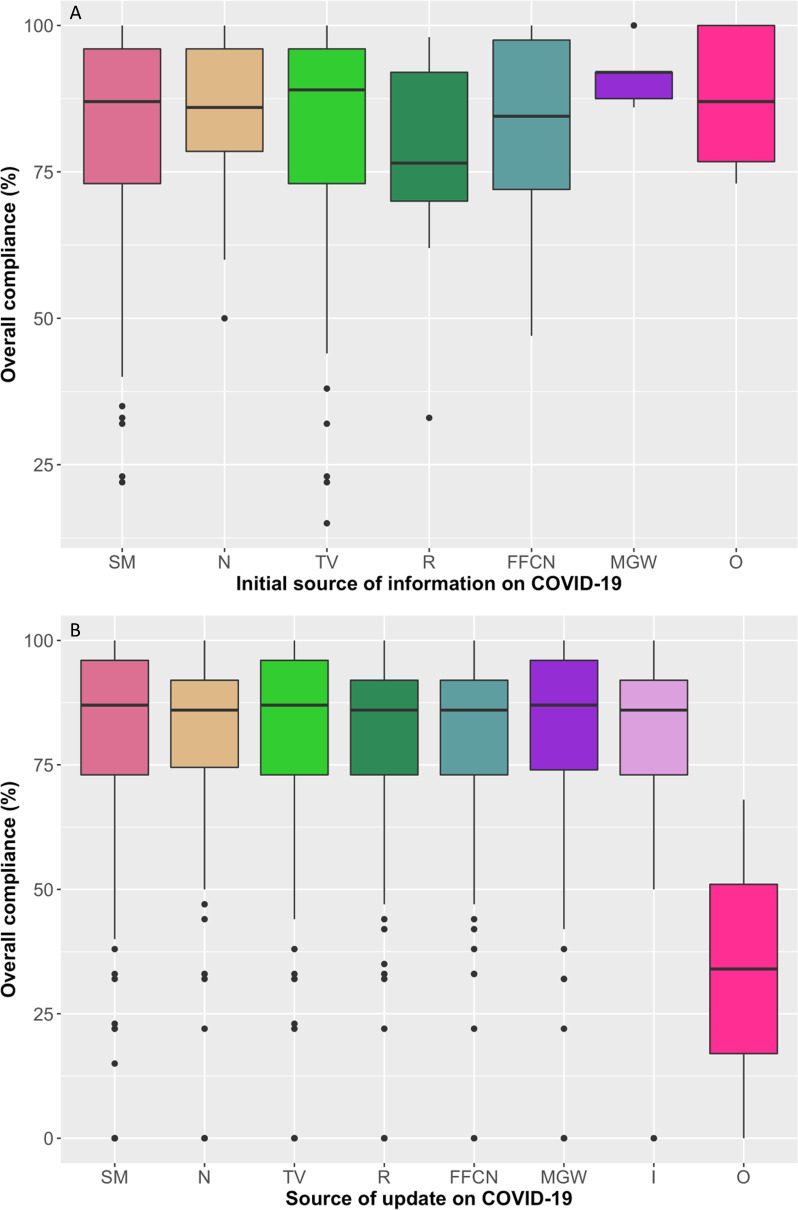
A. Overall compliance of respondents disaggregated by the source of media from which respondents initially heard about COVID-19 B. Overall compliance of respondents disaggregated by sources of media used by respondents to continue to stay up to date about COVID-19. Abbreviations: SM = social media, N = newspaper, TV = television, R = radio, FFCN = family friends colleagues or neighbours, MGW = Ministry of health or Government of Kingdom of Eswatini websites, O = other, I = internet.

Most respondents first heard about COVID-19 from social media (46%) and television (33%), and continued to use these as their source of information to stay updated (Fig 6A & 6B), see questions 12 and 13 in S1 Text. The chi-square test of independence showed that there was no association between initial sources of information with beliefs of COVID-19. However, there was a significant positive association between those who stayed up to date using the radio (χ^2^ = 10.803, d.f. = 3, p<0.005), those who did not indicate their beliefs about COVID-19 (χ^2^ = 10.05, d.f. = 1, p<0.01), and those who believed that COVID-19 kills “only Americans, Chinese and Europeans” (χ^2^ = 9.135, d.f. = 1, p<0.01). The effect size (Cramer’s V) was moderate (0.22), weak (0.15) and weak (0.14), respectively.

According to the chi-square test of independence, there was no significant difference between males and females in their choice of media to continue staying updated about COVID-19 (Fig C in S5C–S5D Fig). Social media was the preferred media used by 18–44 year olds to stay updated about COVID-19, whilst television was preferred by those 45 years and above (Fig D in S5C–S5D Fig).

Respondents who were least compliant to government public health and social measures first learnt about COVID-19 from social media (average overall compliance = 82.1%), family members, friends, colleagues or neighbours (average overall compliance = 82.5%), television (average overall compliance = 82.7%) and radio (average overall compliance = 77.2%), and continued to use all forms of media for information about COVID-19, Fig 6A and 6B. The chi-square test of independence showed that there was no association between overall compliance and media source (initial or continued) used for COVID-19 (p>0.05, for all cases). However, there were significant positive associations between compliance to personal protective measures and those who stayed up to date with information on COVID-19 through the internet (p<0.001), social media (p<0.01), newspapers (p<0.01) and Ministry of Health and Government of Kingdom of Eswatini websites (p<0.01), and effect size (Cramér’s V) of these associations were found to be weak, except the internet which was moderate (S1 Table). There was no significant association between activities carried out whilst away from home and travel compliance with sources of media used to stay up to date about COVID-19 (p>0.05, for all cases), as shown in S1 Table.

### Analysis of missing data

A sensitivity analysis using multinomial logistic regression and predictive mean matching was carried out (S6 Fig) [68]. Approximately 8% of the survey data was missing therefore eight imputations were carried out as part of the analysis. The data from each of the eight imputes were reanalysed using stepwise backward generalised linear model with gamma distribution. The best performing models again included public transport (for 6 out of 8 imputes), and gender (1 out of 8 imputes) as significant variables for overall compliance. Additionally, age was found to be significantly associated in one of the eight imputes. However, essential workers were not a significant variable. Therefore, we conclude that our survey data was overall robust, but data from essential workers may not be robust and additional studies such as a survey aimed at essential workers may be required to better understand this group.

## Discussion

The Government of Eswatini introduced public health and social measures to control the spread of COVID-19 disease [40, 44, 45, 69]. This study found adult internet users to be largely compliant with these measures with an average overall compliance of 73% (SD = 31%), 90% of respondents perceived COVID-19 to be a serious threat, and the availability of PPE for essential workers to be a challenge. Social media, television and government websites were continued sources of COVID-19 information and should be used to reach this target population in Eswatini. The demographics of our survey participants was in line with what we would expect of internet users living in Eswatini.

This study found a variety of variables that significantly influenced compliance including gender, age using public transport, and beliefs surrounding COVID-19. Gender differences in compliance has been found in other studies, where males were significantly associated with risky behaviours related to COVID-19 (e.g., going to crowded places, not wearing a mask) [16]. This study found females reported being significantly (p<0.001) more compliant than males to government public health and social measures, particularly with protecting themselves from COVID-19 and following the government’s travel advice [44]. This could be due to social and cultural differences (e.g. polygamy), as males in Eswatini are often responsible for multiple households and work away from home. This could also be due to social desirability bias in females [70]; however, women were also significantly found to be more likely to comply with COVID-19 preventive measures early in the pandemic, in online surveys in other countries such as Qatar [18], Pakistan [19], Ecuador [22] and eight OECD countries where social desirability bias was controlled [20]. Other studies have shown more women tend to actively seek health-related information than men [71–75] and engage in less risky behaviour [76–80]. One Finnish study demonstrated females were significantly more interested in health-related information and attentive to potential worldwide pandemics [71].

Age differences in compliance with COVID-19 prevention measures has been shown in the U.S. [21], Ecuador [22] and Pakistan [19] with younger adults least likely to be compliant. However, Zhong et al. [16] did not find age to be a significant determinant of risky behaviours towards COVID-19. In this study, overall compliance significantly increased with age (p<0.01), and the youngest group age 18–29 were less compliant with personal protection and the types of activities carried out whilst away from home. There was no significant difference with age for travel compliance. This could be due to social desirability bias as young adults tend to be less cautious with sharing information [70]; however other studies have shown increased age to be predictor of protective health behaviours [81], with young adults less likely to consider the consequences of their behaviour on their health, and therefore more willing to engage in risky behaviour [81–84]. One Finnish study demonstrated 18–35 year olds to be least attentive to health information related to potential worldwide pandemics [71].

Public transport, especially when crowded, can be associated with the spread and acquisition of respiratory infectious diseases [29, 30]. At the beginning of the crisis, Wuhan shut its public transport system. As the disease spread internationally, other countries such as India, Brazil and Morocco followed suit. Eswatini, UK [31], Peru, Mexico, and Kenya require passengers to wear face coverings, staggering timings and/or a decrease in occupancy [33]. Some countries (e.g., France, Italy and UK) have taken the opportunity to incentivise cycling to reduce pollution and pressure on the public transport system, whilst other countries such as South Africa banned cycling [85, 86]. Our study showed public transport users to be significantly (p < 0.001) less compliant to overall public health and social measures against the spread of COVID-19 and in all three categories (personal protection, activity and travel), however, government guidance of one vacant seat between passengers was observed on public transport by over 80% of users. One reason we postulate public transport users to be less compliant with public health and social measures could be due to increased normalisation of COVID-19 risks-taking behaviours, as they travelled frequently for semi-essential and non-essential activities. There was no significant correlation between public transport users and essential workers; indicating respondents who used public transport were not travelling frequently because of essential work purposes.

Among the many public health and social measures implemented to stop the spread of SARS-COV-2 by governments across the world was for people to stay at home and work from home where possible, with the exception of essential workers who were needed to provide medical care, food and other essential services to the population [12]. In Eswatini, essential workers consisted of a variety of sectors including health, agriculture, financial services, media and telecommunication services [36, 37]. Compliance of essential workers to public health and social measures is particularly important because they can interact with many people in a day thereby inadvertently pick up and unknowingly spreading the virus [87]. In our study, essential workers reported to be significantly more compliant to personal protective and travel measures compared to non-essential workers. This could be due to workplaces being forced to implement protective measures (e.g., social distancing and mask wearing), or otherwise face closure [88]. Few essential workers reported staying at home as a personal protective measure which is expected given their role as essential frontline workers. Over a quarter of essential workers reported to have travelled to village homesteads in the past one month, indicating a potential route for the virus to spread from cities to rural communities. Furthermore, 14.7% of essential workers did not answer the compliance questions, and more than half of them were from the medical and health sector. Not answering questions could be due to a variety of factors including inadvertently missing the question, unwillingness to share due to perceived risks or mistrust in the confidentiality of their responses, lack of motivation to answer questions, and/or poor internet connectivity [89]. However, why it disproportionately affected healthcare workers is not clear and may need further investigation. This survey also asked essential workers how protected they felt from contracting COVID-19, and if they had the necessary PPE. Over 50% of the essential workers felt protected and had the necessary PPE, which they did not share; however, over 30% of essential workers in this survey either shared or had no PPE. Access to PPE, as well as mental and physical stress, were recently highlighted as some of the key challenges faced by essential workers in Africa early on during the COVID-19 pandemic [90]. Further studies with essential workers as the target population would help better understand the needs, challenges and experiences related to COVID-19 in this key population.

Studies have found the number of people in a household to be positively associated with acquiring COVID-19 [91] and MERS [2]. Epidemic models have shown airborne infectious diseases are more easily spread within households, and contact density is the main driver in epidemic spread [34]. Whilst this study found no significant difference between the number of people in a household and the household compliance to government advice on protective measures, there was a significant positive association between COVID-19 symptoms of participants and those of their household (p<0.001). Building designs in Eswatini typically vary between urban and rural areas. Rural homesteads are normally composed of multiple single room buildings, whilst urban homes normally consist of a single building with one or more rooms. Whilst this survey did not ask participants if they lived in rural or urban areas, or their building design, 16% percent of respondents reported they lived at their rural (village) homesteads (Table 1). Cultural norms such as congregating, socialising, and taking meals together as a household can maintain contact density even in low-density households. The relatively low density of households in our study, was revealed by the significant positive association between household size and number of rooms in the house (Fig 5A), which could be attributed to the comparatively affluent socioeconomic background of our target population (internet users). Furthermore, only 4% of our respondents did not have the ability to isolate a household member to a dedicated room. This becomes particularly important as the national guidelines changed in July 2020 from hospitalizing all cases to hospitalising only moderate to severe cases [92].

While, other studies showed that beliefs and knowledge of COVID-19 were correlated with compliance to preventive measures [15, 16, 21], this study did not find a significant association. This could be due to 90% of respondents perceived COVID-19 to be serious threat as indicated by their belief in that it could kill anyone. One online survey across eight OECD countries demonstrated women were more likely to perceive COVID-19 as a serious health threat than men and were likely to take comply with preventive measures [20]. In our study of internet users, social media (46%) and television (33%) were the primary original and continued sources of information for our study population; however, health information in Eswatini is disseminated through a variety of avenues from national media to rural health motivators. Throughout the country, COVID-19 health messaging leveraged the existing systems developed for HIV and tuberculosis with radio, local community leaders, and community health structures utilised to reach those without the means to access more expensive information sources such as newspaper, television and internet. Our study revealed a significant positive association between respondents who stayed up to date with COVID-19 through the radio and those who did not believe COVID-19 could kill anyone (p<0.01). Therefore, targeted messaging could be prepared for radio listeners to address any misconceptions about COVID-19. Furthermore, as stated previously, our study targeted the portion of the Eswatini population who access the internet, therefore it would be interesting to investigate and compare the compliance, challenges, beliefs and knowledge of members of the public without access to the internet.

## Limitations

This study has limitations that should be considered when interpreting the data. Due to travel restrictions, traditional door-to-door or face-to-face surveys were not employed. The high costs of communication and the lack of available funding for this study made SMS or telephonic surveys prohibitively expensive, and sampling would be restricted to the limited database(s) we could employ. Therefore, this study used an online survey coupled with website, traditional media and social media outreach to recruit participants resident in the four regions of Eswatini. This study targeted adults who use the internet (e.g., at home, offices or through smart phones); the survey was not designed to capture the perceptions, attitudes and behaviour of those who did not use such technology during the time the survey was live and cannot be considered as representative of the general public. As described above, approximately half of the population in Eswatini is thought to have access to the internet and a quarter of the population are active monthly social media users [46]. Although 97% of social media users access the internet using their mobile phones and 99% of the population has a mobile connection, the capacity for internet access by mobile phones varies considerably and there are urban and rural differences with urban areas having better network connection infrastructure and the population with the resources to pay for access [46]. This may be reflected by 16% of our respondents indicating they live at their rural (village) homestead. Additionally, by using the national COVID-19 web platforms as recruitment sources, there may be a portion of the respondents who have a bias toward following and specifically being interested in COVID-19 and who may be more likely to be educated on the topic and/or more likely to comply. There is also a voluntary response bias because participants had to actively follow a link to participate in the survey; therefore, those who participated in the survey may have stronger opinions. Survey questions were phrased to assess compliance against government guidance in place at the time. When the guidance changed on the 1^st^ June 2020, the survey was closed. Therefore, the online survey was live on the SurveyMonkey website for a limited time thereby restricting the number of participants. Our study uses data from respondents self-reporting using an online survey and therefore can be affected by social desirability bias. Whilst this was not controlled for in the study questions, the survey was anonymous, voluntary and respondents could backtrack and change their answers before submitting their survey, which has been associated with a decrease in social desirability bias [93].

## Conclusions

Our online survey indicates that between 14–31 May 2020, adult residents in the southern African Kingdom of Eswatini with access to the internet were mostly compliant to government public health and social measures. Females, public transport users, older age groups and those with medical aid reported greater compliance to these COVID-19 prevention measures. Essential workers were overall compliant to government measures; however, the provision of dedicated personal protective equipment was highlighted as a challenge for them. Most respondents to our survey had the capacity to isolate household members, which is important as changes to the Government’s testing and treating strategy implemented two months after the close of survey saw persons presumed to have COVID-19 to be isolated at home, and only those with moderate to severe symptoms to be hospitalised.

## Supporting information

S1 Text Survey questions (English). (PDF)

S1B-E Fig B. Compliance of personal protective measures by gender. C. Travel compliance to village homestead by gender. D. Travel frequency compliance by gender E. Travel compliance during public holidays by gender. WHS = washing hands and sanitising. SD = social distancing. SH = staying home. WM = wearing a mask. NOA = none of the above. NSH = no, I stayed home. NVH = no village homestead. LVH = lives at village homestead. Y12 = yes, once or twice. Y3+ = yes 3 or more times. DNA = did not answer.
(TIF)

S2C-G Fig C. Compliance of personal protective measures by essential workers. D. Travel compliance to village homestead by essential workers. E. Travel frequency compliance by essential workers F. Travel compliance during public holidays by essential workers. G. Overall compliance score for essential workers. Abbreviations: FA = food and agriculture, RCGS = retail and consumer goods supplier, NI = network infrastructure, MH = medical and health, FSM = forestry and saw mills, ITST = IT systems and telecommunications, FI = finance and insurance, TH = tourism and hospitality, Comm = communications (e.g., media), HS = hardware stores, NPO = non-profit organisations, Edu = education, O = other, WHS = washing hands and sanitising. SD = social distancing. SH = staying home. WM = wearing a mask. NOA = none of the above. NSH = no, I stayed home. NVH = no village homestead. LVH = lives at village homestead. Y12 = yes, once or twice. Y3+ = yes 3 or more times. DNA = did not answer, Yes = essential worker (n = 129), No = not essential worker (n = 328).
(TIF)

S3C-F Fig C. Compliance of personal protective measures by public transport users. D. Travel compliance to village homestead by public transport users. E. Travel frequency compliance by public transport users F. Travel compliance during public holidays by public transport users. Abbreviations: WHS = washing hands and sanitising. SD = social distancing. SH = staying home. WM = wearing a mask. NOA = none of the above. NSH = no, I stayed home. NVH = no village homestead. LVH = lives at village homestead. Y12 = yes, once or twice. Y3+ = yes 3 or more times. DNA = did not answer. Yes = public transport user (n = 102), No = not public transport user (n = 302). Fifty-three respondents declined to answer if they did or did not use public transport.
(TIF)

S4B-E Fig B. Compliance of personal protective measures by age C. Travel compliance to village homestead by public transport users. D. Travel frequency compliance by public transport users E. Travel compliance during public holidays by public transport users. Abbreviations: WHS = washing hands and sanitising. SD = social distancing. SH = staying home. WM = wearing a mask. NOA = none of the above. NSH = no, I stayed home. NVH = no village homestead. LVH = lives at village homestead. Y12 = yes, once or twice. Y3+ = yes 3 or more times. DNA = did not answer. One respondent declined to identify their age.
(TIF)

S5C-D Fig C. Sources of media used to continue staying up to date about COVID-19, by gender. D. Sources of media used to continue staying up to date about COVID-19, by age. SM = social media, N = newspaper, TV = television, R = radio, FFCN = family friends colleagues or neighbours, MGW = Ministry of health or Government of Kingdom of Eswatini website, O = other, I = internet.
(TIF)

S6 Fig Distribution of imputed and observed values for overall compliance. Light grey = observed values. Imputed values = dark grey.
(TIF)

S1 Table Association of respondents’ compliance to protection, activity and travel measures with media source used to stay up to date about COVID-19. (PDF)
