# Supplementary material for: Assessment of early COVID-19 compliance to and challenges with public health and social prevention measures in the Kingdom of Eswatini, using an online survey
Source: PLoS One. 2021 Jun 29;16(6):e0253954. doi: 10.1371/journal.pone.0253954 (PMC8241123; doi:10.1371/journal.pone.0253954)
Supplement: S1 Text — (PDF) [file pone.0253954.s001.pdf]

Survey Questions (English)

1. Have you previously completed this survey?

    - Yes
    - No

2. What is your age?

    - 18-29
    - 30-44
    - 45-59
    - 60 or older

3. What is your gender?

    - Female
    - Male

4. In which region of Eswatini do you live?

    - Hhohho
    - Lubombo
    - Manzini
    - Shiselweni
    - Outside Eswatini

5. How many people currently live in your household?

    - 1 (I live alone)
    - 2 – 3
    - 4-5

- 6 or more

6. How many rooms (excluding kitchen and bathroom) do you have in your house?

   - 0-1 (e.g. bedsit)
   - 2-3
   - 4-5
   - 6 or more

7. Do you currently have medical aid (e.g. private medical insurance)?

   - Yes
   - No

8. What is the highest level of school that you have completed?

   - Less than primary school
   - Primary school
   - Some high school, but no certificate
   - High school
   - Some college or university, but no qualification
   - College or university qualification (e.g. Certificate, Diploma, Bachelors)
   - Post-graduate-level qualification(s) (e.g. Masters, PhD)
   - None of the above

9. Are you an essential service worker?

   - Yes
   - No

If the respondent answers yes to question 9 then questions 10 & 11 apply:

10. What essential service are you employed in?

- Food and Agriculture
- Retail and Consumer Goods Supplier (e.g. food retail; consumer goods; transport, logistics and packing of consumer goods; retails goods for medical industry)
- Network infrastructure (Water, waste water and sanitation; energy and fuel supply; law enforcement; defence, safety and security; waste disposal; fire and emergency services; mining and power stations)
- Medical and Health (hospitals, devices and equipment, pharmaceuticals, funders, healthcare workers)
- Forestry and saw mills (for production of disposable, health and hygiene products)
- IT Systems and Telecommunication
- Finance and Insurance
- Tourism and Hospitality
- Communications
- Hardware shops
- Public Transport

11. As an essential worker, do you feel protected from contracting the virus?

- Yes, we have been given the necessary personal protective equipment which we **do not** share with other staff
- Yes, we have been given the necessary protective equipment which we **do** share with other staff
- Yes, I do not have contact with anyone
- No, I have not been given the necessary protective equipment

12. How did you first learn about coronavirus (COVID-19)?

- Social media
- Newspaper
- Television
- Radio
- Family member, friend, colleague or neighbour
- Ministry of Health or Government of Kingdom of Eswatini website
- Other (please specify)

13. Which of the following are you using to stay updated on COVID-19 (tick all that apply)?
    - Social media
    - Newspaper
    - Television
    - Radio
    - Family members, friends, colleagues or neighbours
    - Ministry of Health or Government of Kingdom of Eswatini website
    - Other (please specify)

14. What do you believe COVID-19 is?
    - A disease that can kill anyone
    - A disease that is killing only Europeans, Chinese and Americans
    - It does not exist

15. Which of these symptoms have you experienced in the past two weeks?
    - Fever
    - Dry cough
    - Tiredness

- Shortness of breath
- other flu-like symptoms
- None of the above

16. Has anyone else in your household experienced any of these five symptoms in the past two weeks?
    - Fever
    - Dry cough
    - Tiredness
    - Shortness of breath
    - Other flu-like symptoms
    - None of the above

17. Have you ever been screened or tested for COVID-19?
    - Yes
    - No

    If the respondent answers yes to question 17 then question 18 applies:

18. The results of your COVID-19 test or screening were:
    a. Positive (I had or might have COVID-19)
    b. Negative (I did **not** have COVID-19)
    c. I’m still waiting on the results

19. Have you been in contact with someone who has been diagnosed with COVID-19?
    - Yes
    - No
    - I’m not sure

20. What precautions have you been taking to protect yourself against COVID-19 (tick all that apply)?
    - Washing or sanitizing hands
    - Social distancing (deliberately keeping more than 1m away between you and someone else who does not live with you)
    - Staying home
    - Wearing a mask at all times when away from your home
    - None of the above

21. What precautions are members of your household taking to protect themselves against COVID-19 (tick all that apply)?
    - Washing or sanitizing hands
    - Social distancing (deliberately keeping more than 1m away between you and someone else who does not live with you)
    - Staying home
    - Wearing a mask at all times when away from your home
    - None of the above

22. During the past seven days, how many times did you leave your home?
    - Never
    - Once
    - 1-2 times
    - 3-5 times
    - 6 or more times

23. What are the reasons for leaving your home (tick all that apply)?
    - Work

- Shopping
- Medical reasons
- Visiting other homes
- Other (please specify)

24. How far is your nearest food shopping facility?
    - Very far (5km or more)
    - Not so far (1-5kms)
    - Very close (less than 1km)

25. Do you use public transportation?
    - Yes
    - No

If respondent answers yes to public transport (question 25) – the following question applies:

26. When using public transportation, how full is the vehicle?
    - Very full (no space between passengers)
    - Not full, there is space for at least 1 person between me and the next person

27. In the last one month, have you travelled to your village homestead?
    - Yes, several times (e.g. 3 times or more)
    - Yes, once or twice
    - I live at my village homestead
    - No, I do not have a village homestead
    - No, I stayed at home

28. Over the Easter holidays or recent public holidays, did you travel within the country to visit friends or family?

- Yes
- No
