# Supplementary figures and images for: Assessment of early COVID-19 compliance to and challenges with public health and social prevention measures in the Kingdom of Eswatini, using an online survey
Source: PLoS One. 2021 Jun 29;16(6):e0253954. doi: 10.1371/journal.pone.0253954 (PMC8241123; doi:10.1371/journal.pone.0253954)

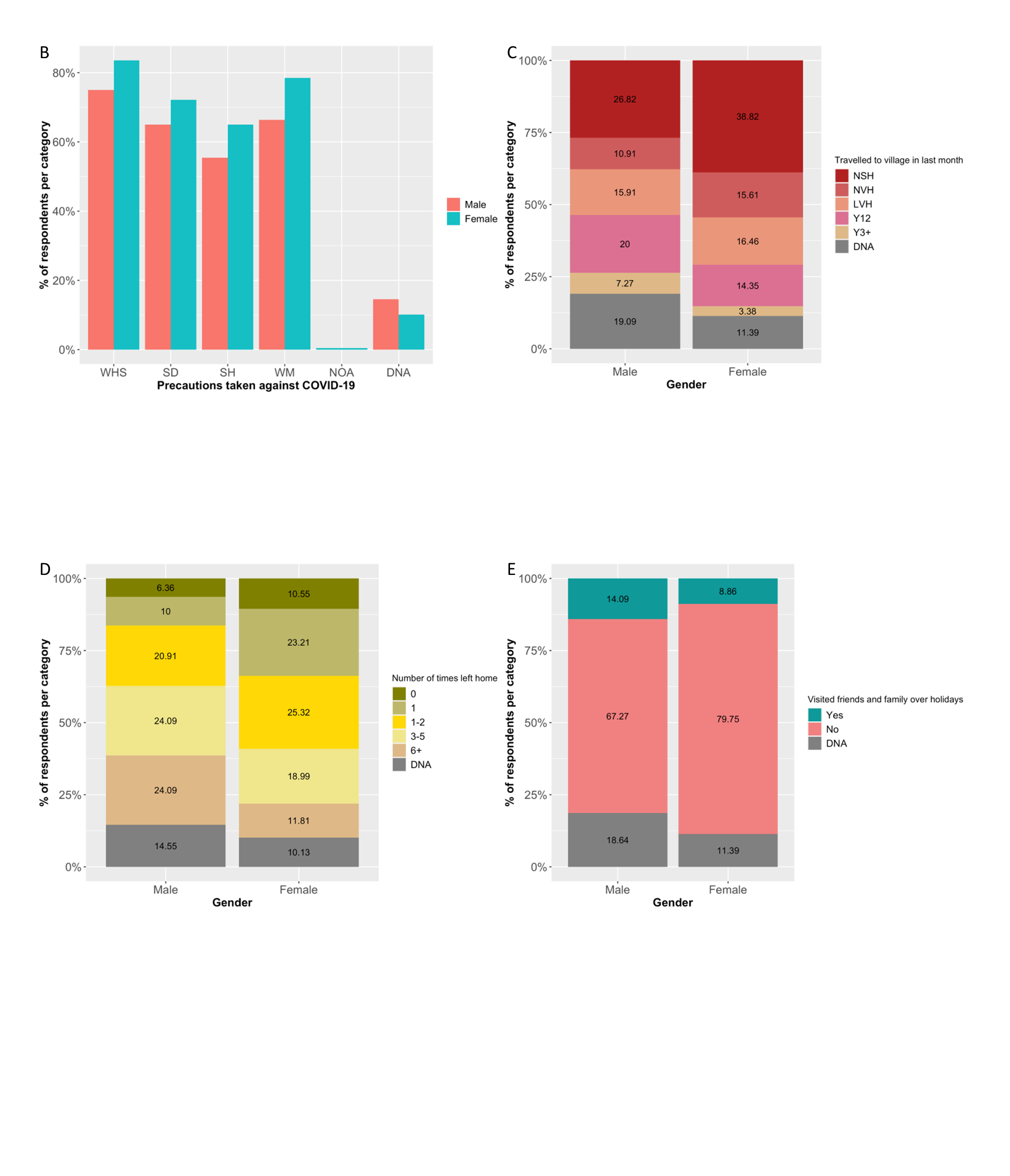

Supplement: S1B-E Fig — B. Compliance of personal protective measures by gender. C. Travel compliance to village homestead by gender. D. Travel frequency compliance by gender E. Travel compliance during public holidays by gender. WHS = washing hands and sanitising. SD = social distancing. SH = staying home. WM = wearing a mask. NOA = none of the above. NSH = no, I stayed home. NVH = no village homestead. LVH = lives at village homestead. Y12 = yes, once or twice. Y3+ = yes 3 or more times. DNA = did not answer. (TIF) [file pone.0253954.s002.tif]

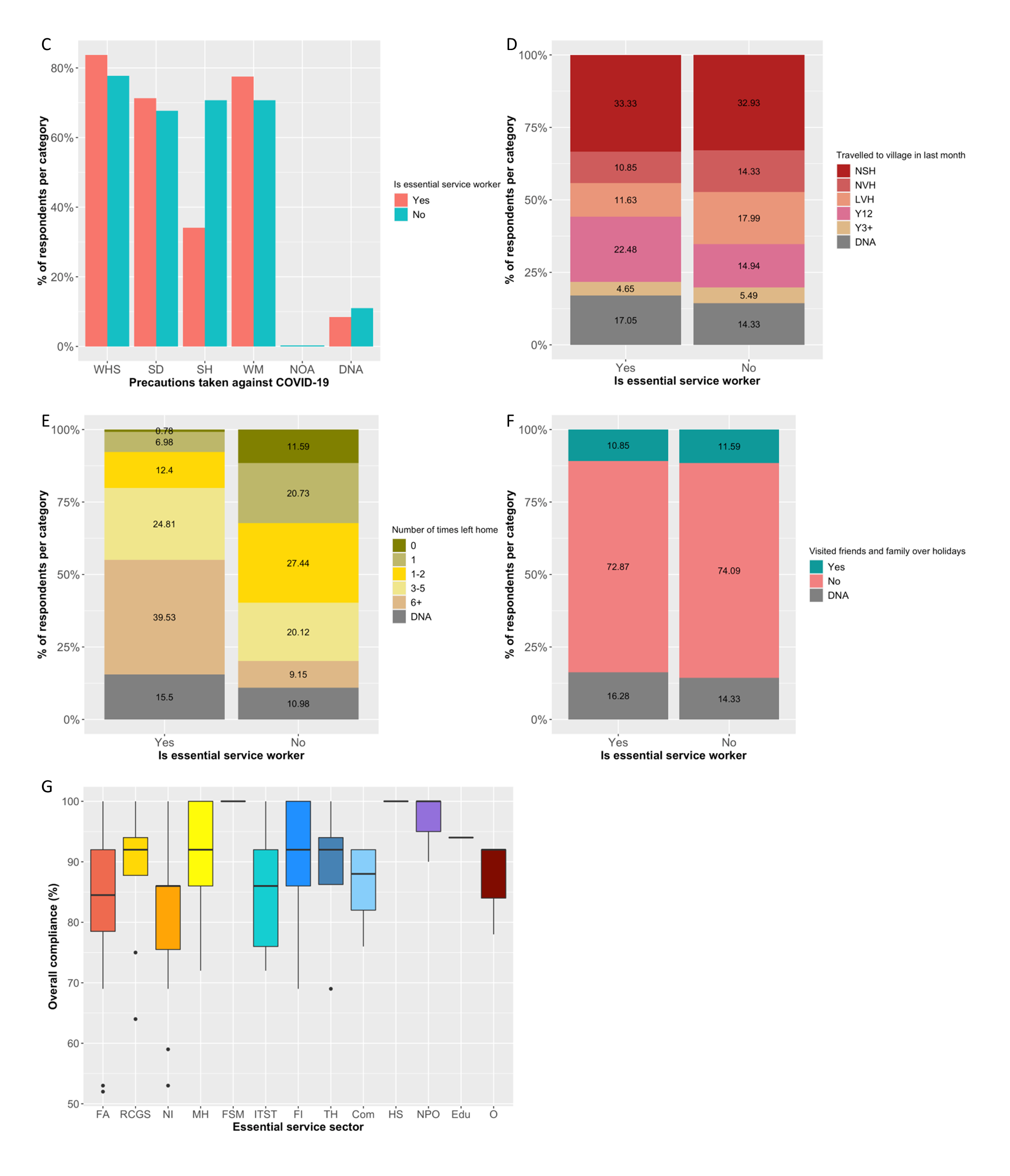

Supplement: S2C-G Fig — C. Compliance of personal protective measures by essential workers. D. Travel compliance to village homestead by essential workers. E. Travel frequency compliance by essential workers F. Travel compliance during public holidays by essential workers. G. Overall compliance score for essential workers. Abbreviations: FA = food and agriculture, RCGS = retail and consumer goods supplier, NI = network infrastructure, MH = medical and health, FSM = forestry and saw mills, ITST = IT systems and telecommunications, FI = finance and insurance, TH = tourism and hospitality, Comm = communications (e.g., media), HS = hardware stores, NPO = non-profit organisations, Edu = education, O = other, WHS = washing hands and sanitising. SD = social distancing. SH = staying home. WM = wearing a mask. NOA = none of the above. NSH = no, I stayed home. NVH = no village homestead. LVH = lives at village homestead. Y12 = yes, once or twice. Y3+ = yes 3 or more times. DNA = did not answer, Yes = essential worker (n = 129), No = not essential worker (n = 328). (TIF) [file pone.0253954.s003.tif]

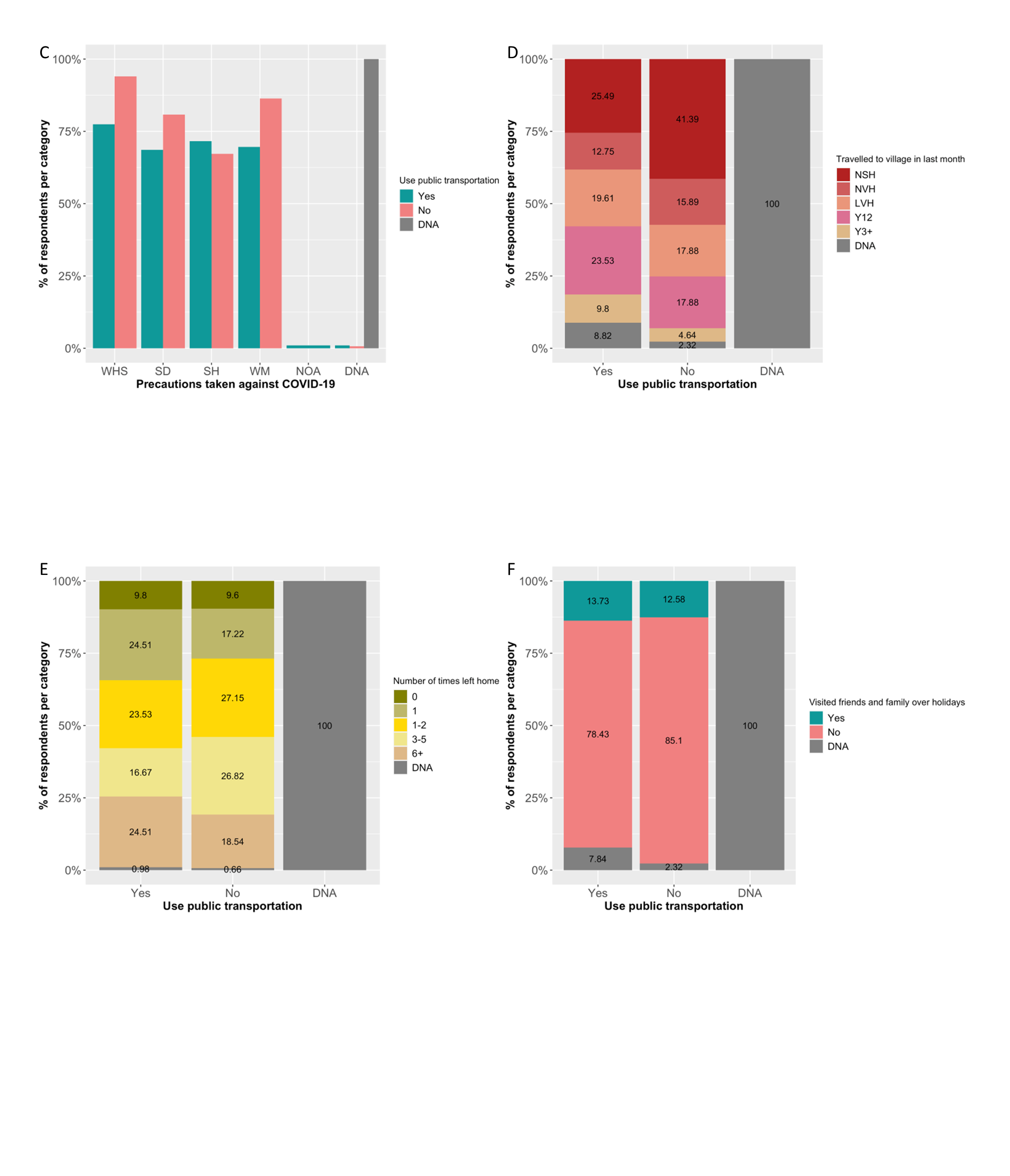

Supplement: S3C-F Fig — C. Compliance of personal protective measures by public transport users. D. Travel compliance to village homestead by public transport users. E. Travel frequency compliance by public transport users F. Travel compliance during public holidays by public transport users. Abbreviations: WHS = washing hands and sanitising. SD = social distancing. SH = staying home. WM = wearing a mask. NOA = none of the above. NSH = no, I stayed home. NVH = no village homestead. LVH = lives at village homestead. Y12 = yes, once or twice. Y3+ = yes 3 or more times. DNA = did not answer. Yes = public transport user (n = 102), No = not public transport user (n = 302). Fifty-three respondents declined to answer if they did or did not use public transport. (TIF) [file pone.0253954.s004.tif]

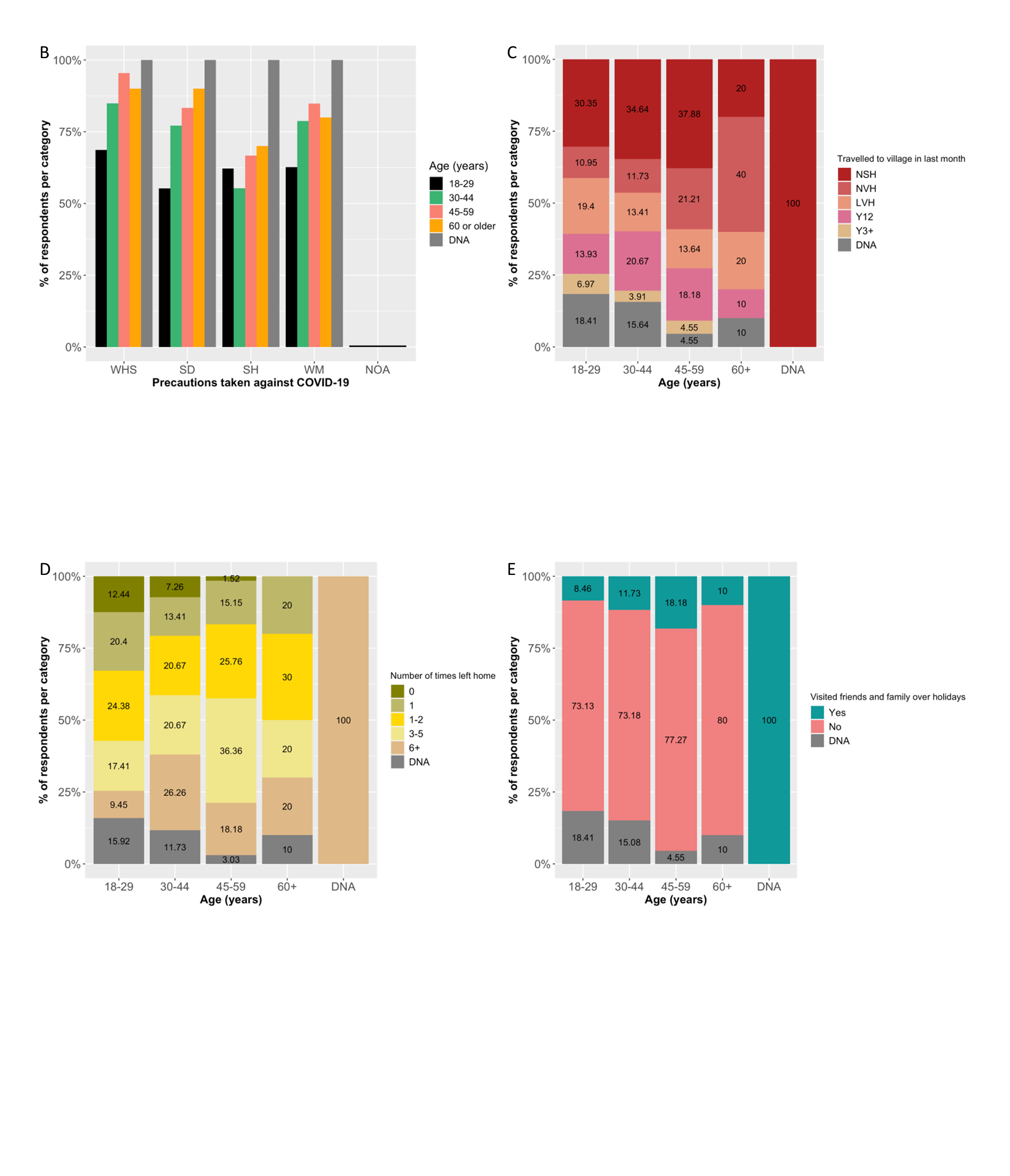

Supplement: S4B-E Fig — B. Compliance of personal protective measures by age C. Travel compliance to village homestead by public transport users. D. Travel frequency compliance by public transport users E. Travel compliance during public holidays by public transport users. Abbreviations: WHS = washing hands and sanitising. SD = social distancing. SH = staying home. WM = wearing a mask. NOA = none of the above. NSH = no, I stayed home. NVH = no village homestead. LVH = lives at village homestead. Y12 = yes, once or twice. Y3+ = yes 3 or more times. DNA = did not answer. One respondent declined to identify their age. (TIF) [file pone.0253954.s005.tif]

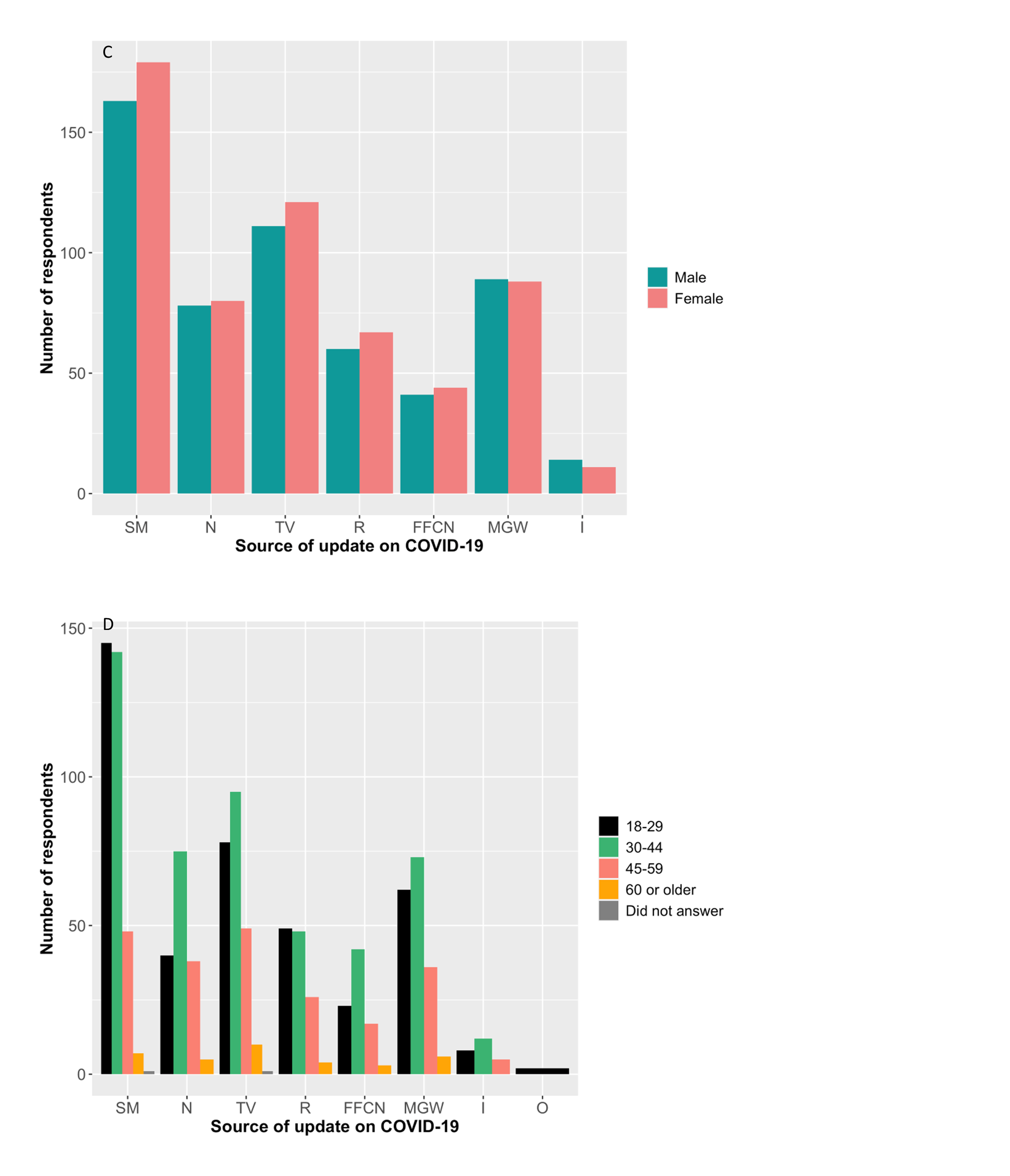

Supplement: S5C-D Fig — D. Sources of media used to continue staying up to date about COVID-19, by age. SM = social media, N = newspaper, TV = television, R = radio, FFCN = family friends colleagues or neighbours, MGW = Ministry of health or Government of Kingdom of Eswatini website, O = other, I = internet. (TIF) [file pone.0253954.s006.tif]

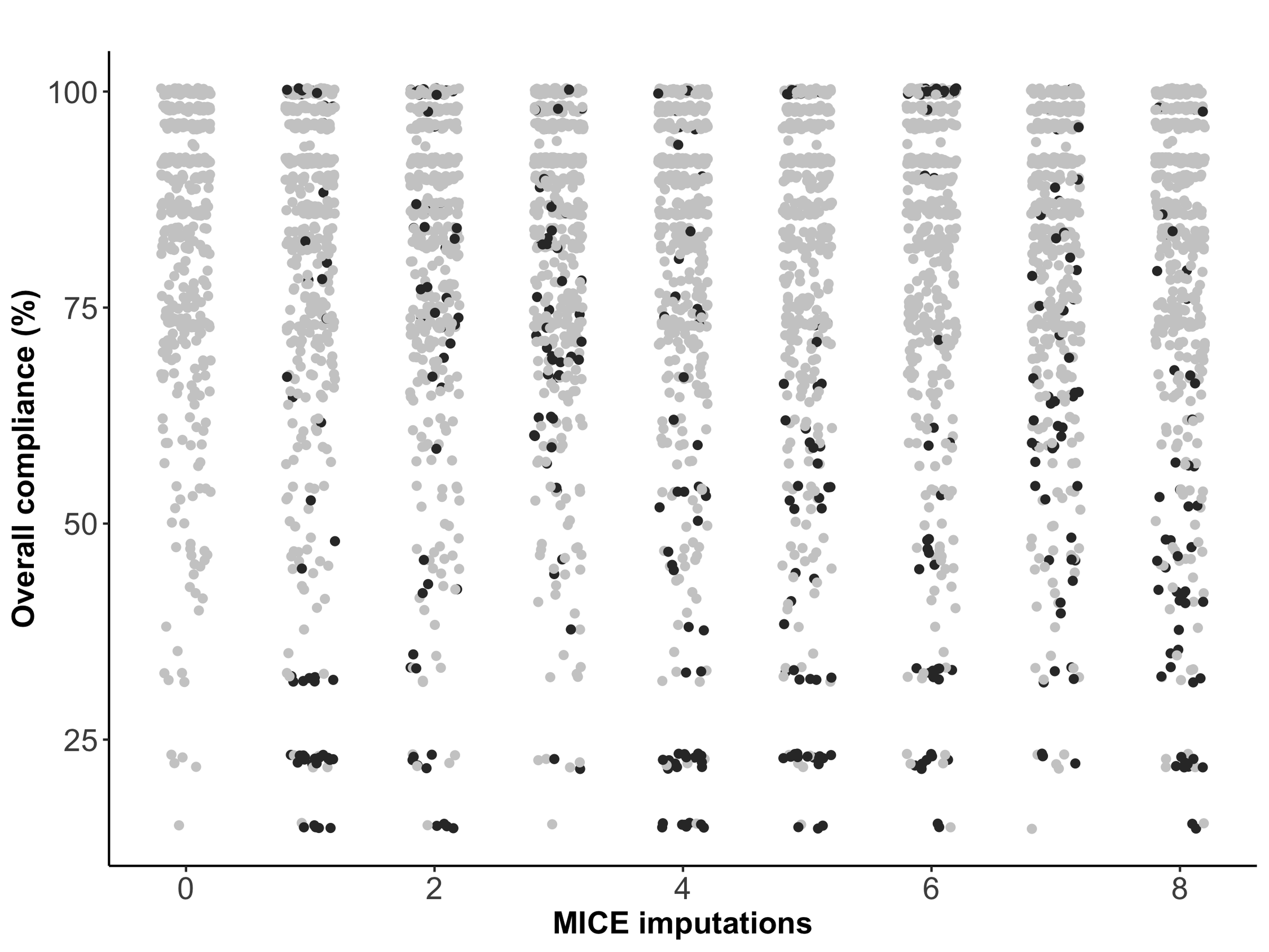

Supplement: S6 Fig — Light grey = observed values. Imputed values = dark grey. (TIF) [file pone.0253954.s007.tif]
