# Supplementary material for: Assessment of early COVID-19 compliance to and challenges with public health and social prevention measures in the Kingdom of Eswatini, using an online survey
Source: PLoS One. 2021 Jun 29;16(6):e0253954. doi: 10.1371/journal.pone.0253954 (PMC8241123; doi:10.1371/journal.pone.0253954)
Supplement: S1 Table — (PDF) [file pone.0253954.s008.pdf]

Table 4 Association of respondents' compliance to protection, activity and travel measures with media source used to stay up to date about COVID-19

Media Source	Chi-square test of independence with Cramer's V											
	Protective measures				Activities				Travel measures			
	χ^2	df	p-value	CV	χ^2	df	p-value	CV	χ^2	df	p-value	CV
**Social Media**	**12.198**	**3**	**0.007**	**0.17**	0.493	2	0.782	0.04	1.079	3	0.782	0.05
**Newspaper**	**12.667**	**3**	**0.005**	**0.18**	0.470	2	0.791	0.03	5.360	3	0.147	0.12
Television	7.664	3	0.054	0.14	0.243	2	0.886	0.02	2.138	3	0.544	0.07
Radio	5.049	3	0.168	0.11	3.720	2	0.156	0.10	0.147	3	0.986	0.02
Friends, family, neighbours & colleagues	1.257	3	0.740	0.06	3.094	2	0.213	0.09	3.614	3	0.306	0.09
**Ministry of Health & Government websites**	**14.720**	**3**	**0.003**	**0.19**	1.850	2	0.397	0.07	1.850	3	0.489	0.08
**Internet (e.g., online news)**	***17.424***	***3***	***0.001***	***0.21***	*3.636*	*2*	*0.162*	*0.1*	*1.477*	*3*	*0.656*	*0.06*

Significant media sources highlighted in **bold,** df = degrees of freedom, CV = Cramer's V.
